# Physics-Guided Bioinspired Multi-Objective Optimization for Explainable Drying-Stage Identification in an R290/R600a Waste-Heat Drying System

**DOI:** 10.3390/biomimetics11100749

**Published:** 2026-10-16

**Authors:** Mehmet Das, Suna Yildirim, Oguzhan Pektezel, Bilal Alatas, Ebru Akpinar

**Affiliations:** 1 Department of Mechanical Engineering, Faculty of Engineering, Firat University, 23119 Elazig, Türkiye; m.das@firat.edu.tr (M.D.); ebruakpinar@firat.edu.tr (E.A.); 2 Department of Software Engineering, Faculty of Engineering and Natural Sciences, Malatya Turgut Özal University, 44920 Malatya, Türkiye; suna.yildirim@ozal.edu.tr; 3 Mechanical Engineering Department, Balikesir University, 10145 Balıkesir, Türkiye; 4 Department of Software Engineering, Faculty of Engineering, Firat University, 23119 Elazig, Türkiye; balatas@firat.edu.tr

**Keywords:** R290, R600a, waste-heat drying, bioinspired evolutionary algorithm, multi-objective optimization, explainable artificial intelligence, ordinal classification, P-CRb-SPEA2

## Abstract

Integrating low-global-warming-potential refrigerants, waste-heat recovery, and intelligent process monitoring offers a promising route to energy-efficient drying systems. This study experimentally compares the natural refrigerants R290 and R600a in a vapor-compression refrigeration system and utilizes condenser waste heat for apple drying. Refrigeration tests were conducted at an evaporator temperature of −16 °C and condenser temperatures of 25, 30, 35, and 40 °C. R290 provided 73% higher cooling capacity and 59% greater condenser heat rejection than R600a, while reducing the drying time from 300 to 265 min. Conversely, R600a exhibited lower specific energy consumption and a higher specific moisture extraction rate. To identify drying progress without continuous product weighing, a physics-guided bioinspired multi-objective evolutionary algorithm, termed Physics-Guided Ordinal Chaotic Rule-Based SPEA2 (P-CRb-SPEA2), was developed. The proposed framework integrates Pareto-based evolutionary search, chaotic initialization, ordinal error, physical consistency, and rule complexity to extract interpretable IF–THEN rules from six thermal and environmental features. Ten-fold cross-validation showed physically consistent, interpretable drying-stage classification, with T_10_ and T_8_ emerging as dominant features for R290 and R600a, respectively. The proposed approach demonstrates the potential of bioinspired evolutionary intelligence for explainable monitoring of integrated refrigeration–drying energy systems.

## 1. Introduction

Vapor compression refrigeration systems are used in various fields, including food preservation in domestic conditions, large-scale industrial food storage applications, and air conditioning applications to provide thermal comfort conditions in places. The existence of refrigeration systems is very important, especially for pharmacies, valuable goods, and food transporters. Although there are various methods to achieve the required cooling in these mentioned sectors, most of the cooling systems work with the vapor compression principle. It is forecasted that approximately 1.5 billion household refrigerators are in operation in today’s world, and it is also predicted that 17% of the world’s electricity consumption is spent in the refrigeration sector and 45% of this 17% portion is used in residential applications [1]. The refrigerant market, valued at 21.3 billion dollars in 2020, is anticipated to expand to approximately 30.8 billion dollars by the year 2025 [2]. Conventional air conditioning and refrigeration systems consume substantial energy through the compressor and contribute to the greenhouse effect [3]. Considering the effects of old-generation refrigerants on the global warming potential of the world, the heat pump and refrigeration sectors have tried to discover new refrigerants with low GWP [4]. Recently, R290, R1234yf, and R600a have attracted growing attention owing to their very low global warming potential and favorable thermodynamic properties [5,6].

Various approaches have been suggested to improve the energy performance and efficiency of refrigeration systems, such as improvements in heat exchangers, use of alternative refrigerants, commissioning of high-efficiency compressors, use of improvements in insulation processes, and application of modified refrigeration cycles. It is known that the use of vapor compression refrigeration systems has become widespread in many areas in the world, and energy consumption has increased in parallel.

Some studies have been conducted to detect performance indicators such as compressor power consumption and COP of refrigeration systems operating with low GWP refrigerants. In these studies, new-generation refrigerants caused a decrease in compressor power consumption compared to the old-generation refrigerants and provided a higher COP value in the refrigeration system [7,8,9,10] (Table 1).

Besides their improvements in performance parameters, the effects of low GWP refrigerants on global warming are close to zero, and their ozone depletion potential is zero. In addition, natural refrigerants have been used for over a century as an effective solution for cooling systems [19].

In conventional hot-air drying systems, heat is typically supplied using electricity, LPG, natural gas, or fossil fuels. This not only increases operating costs but also contributes to greater environmental impacts. Particularly in food-drying applications conducted within low- and medium-temperature ranges, the utilization of waste heat offers significant potential for energy savings. Waste heat-assisted drying systems are based on the utilization of heat released from an existing process or energy conversion system to heat the drying air. This approach can reduce the primary energy demand required for drying and thereby improve the overall energy efficiency of the system [20,21].

Heat pump drying systems play an important role among energy-efficient drying technologies. In these systems, energy extracted from a low-temperature heat source is transferred to a higher temperature level through a vapor-compression cycle consisting of an evaporator, compressor, condenser, and expansion device. The heat released by the condenser can be utilized to heat the drying air. Chua et al. [22] reported that heat pump drying systems offer advantages over conventional dryers in terms of temperature and humidity control, providing significant benefits for preserving product quality, particularly in the drying of heat-sensitive materials. Similarly, Colak and Hepbasli [23] stated that heat pump drying systems have the potential to reduce energy consumption and provide better control of drying conditions for different products.

Studies on heat pump-assisted systems for drying agricultural products have demonstrated that these systems not only provide energy savings but can also positively affect product quality by creating a more controlled drying environment [11,12] (Table 1).

The utilization of condenser waste heat from refrigeration systems in drying applications represents a promising heat recovery option, particularly for cold storage facilities, air-conditioning systems, food preservation systems, and commercial refrigeration units. In such systems, while the desired cooling effect is simultaneously provided, the heat rejected by the condenser can be transferred to the drying air. Thus, two different useful processes can be achieved with the same energy input. The literature reports that utilizing waste heat from refrigeration systems for drying purposes can reduce energy costs, improve the overall system efficiency, and provide a sustainable alternative, particularly for agricultural product drying applications [24,25].

Artificial intelligence and machine-learning techniques have increasingly been employed in drying studies to model complex relationships among operating conditions, product characteristics, drying kinetics, and energy-related parameters. Earlier studies demonstrated that artificial neural networks (ANNs) could successfully predict moisture content (MC), moisture ratio (MR), and drying rate (DR) [13]. More recent studies have extended this approach by using support vector regression (SVR), k-nearest neighbors (k-NN), random forest (RF), adaptive neuro-fuzzy inference systems (ANFIS), deep neural networks, and hybrid models [14,15,16,17,18]. Overall, the literature demonstrates that AI-based models can accurately predict drying parameters such as MR, MC, DR, drying time, energy consumption, and product-quality indicators; however, most reported approaches primarily focus on numerical prediction accuracy and generally behave as black-box models with limited consideration of the physical consistency and ordered progression of the drying process. The key findings of the studies reviewed in this section are summarized in Table 1.

Unlike conventional AI-based drying studies that primarily predict continuous MR or MC values, the present study introduces a physics-guided, explainable ordinal classification framework to identify the progression of the drying process without continuous product weighing. The proposed P-CRb-SPEA2 approach classifies the drying process into ordered Early, Intermediate, and Advanced stages based on thermal and environmental measurements obtained from the refrigeration–drying system. Rather than optimizing prediction accuracy alone, the model simultaneously considers recall, ordinal error, physical consistency, and rule complexity within a Pareto-based multi-objective framework. In addition, the method generates sparse and interpretable IF–THEN rules and explicitly incorporates the monotonic physical behavior of process variables into the optimization procedure. The model is applied separately to R290 and R600a datasets, enabling the dominant sensor variables and stage-dependent physical signatures associated with each refrigerant to be identified. Therefore, the novelty of this study lies not only in integrating refrigeration and condenser waste-heat-assisted drying, but also in combining this system with a physics-guided explainable artificial-intelligence framework capable of providing interpretable and physically consistent drying-stage identification.

**Figure 1:**
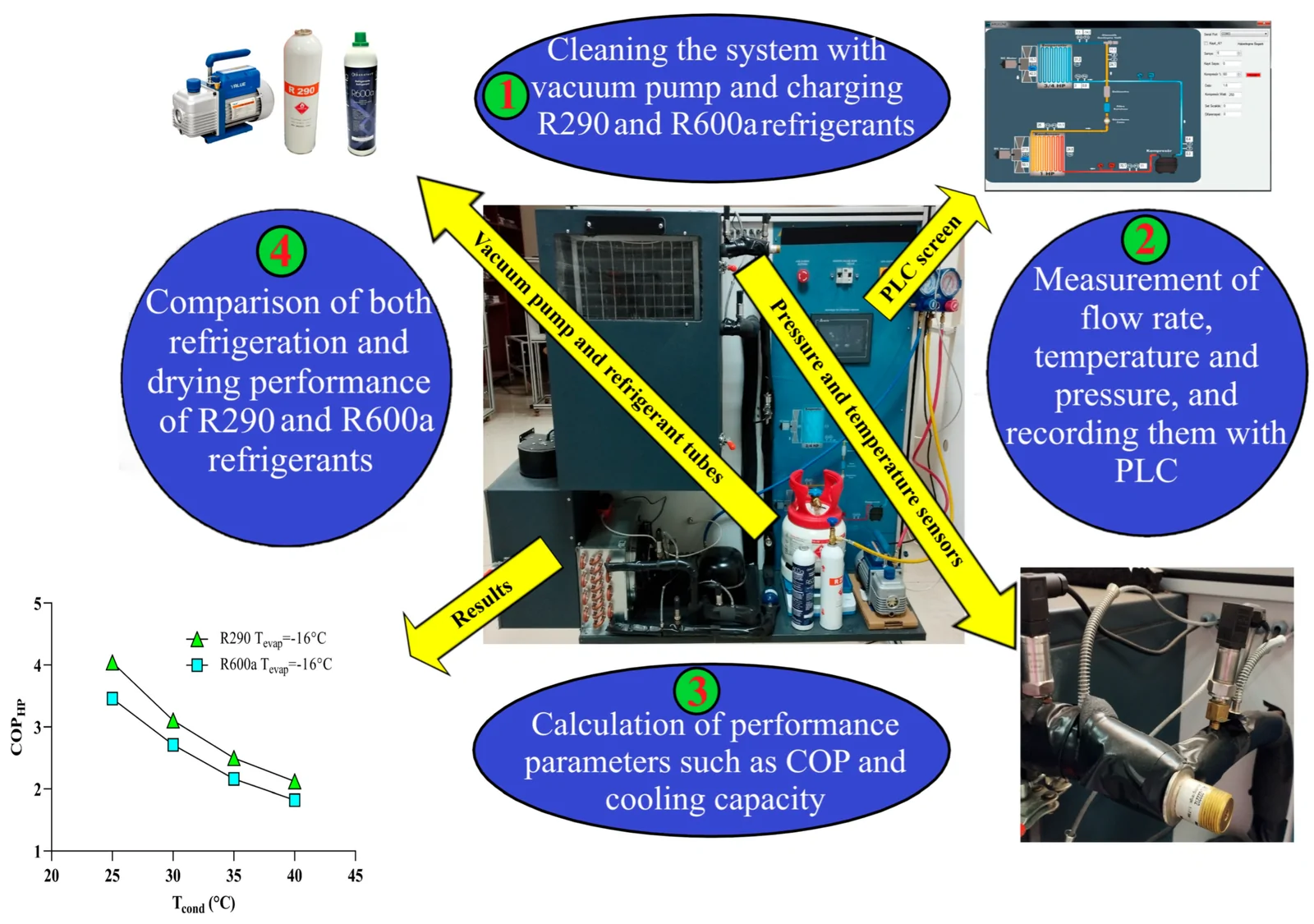
Flow chart of this work.

Figure 1 shows the refrigeration test device in which the experiments were carried out and a summary of the processes performed. Before starting the experiments, the system was vacuumed by means of a vacuum pump and made ready for the gas charging process; firstly, R290 gas was injected into the system. After obtaining data such as temperature, pressure, and flow rate under various operating conditions, R290 gas was evacuated from the system, and vacuum operation was performed again. Afterwards, R600a gas was charged into the system, and the experiments were repeated under the same operating conditions, and the necessary measurement data were recorded with a data logger. The performance of refrigerants was compared in detail with the data obtained.

The primary aim of this study is to evaluate the potential to recover condenser waste heat from a vapor-compression refrigeration system for food drying, while experimentally comparing the performance of the natural hydrocarbon refrigerants R290 and R600a. The main highlights and novelties of the study are summarized as follows:

- Normally rejected condenser heat was converted into a useful thermal source for drying, improving the overall energy utilization of the refrigeration system.
- R290 and R600a were tested separately under different operating conditions, and their refrigeration, drying, and energy performances were comparatively assessed using moisture content, moisture ratio, drying rate, effective moisture diffusivity, activation energy, specific energy consumption, moisture extraction rate, and specific moisture extraction rate, providing practical guidance for the selection of low-GWP refrigerants.
- A physics-guided, explainable artificial intelligence approach was introduced to identify the progression of the drying process from thermal and environmental measurements, reducing dependence on continuous product weighing.
- The proposed framework accounts for the ordered nature of drying stages and incorporates physical consistency into the learning process, enabling the evolution of drying to be interpreted in a manner consistent with process behavior.
- Natural-refrigerant performance assessment, condenser waste-heat recovery, drying kinetics, energy evaluation, and physics-guided intelligent drying-stage identification were combined within a single experimental framework.

This integrated approach provides a broader understanding of the trade-offs among refrigeration performance, drying intensity, energy efficiency, and process-state identification, while offering a practical basis for developing energy-efficient, intelligently monitored refrigeration–drying systems.

## 2. Materials and Methods

This section describes the experimental test bench, the control strategy of the operating conditions, and the thermodynamic and uncertainty analyses used to evaluate the refrigeration performance of R290 and R600a. The drying procedure and the drying-kinetics calculations are then presented, followed by the physics-guided explainable artificial intelligence framework developed for drying-stage identification.

### 2.1. Experimental Test Bench and Control Strategy of Operational Conditions

**Figure 2:**
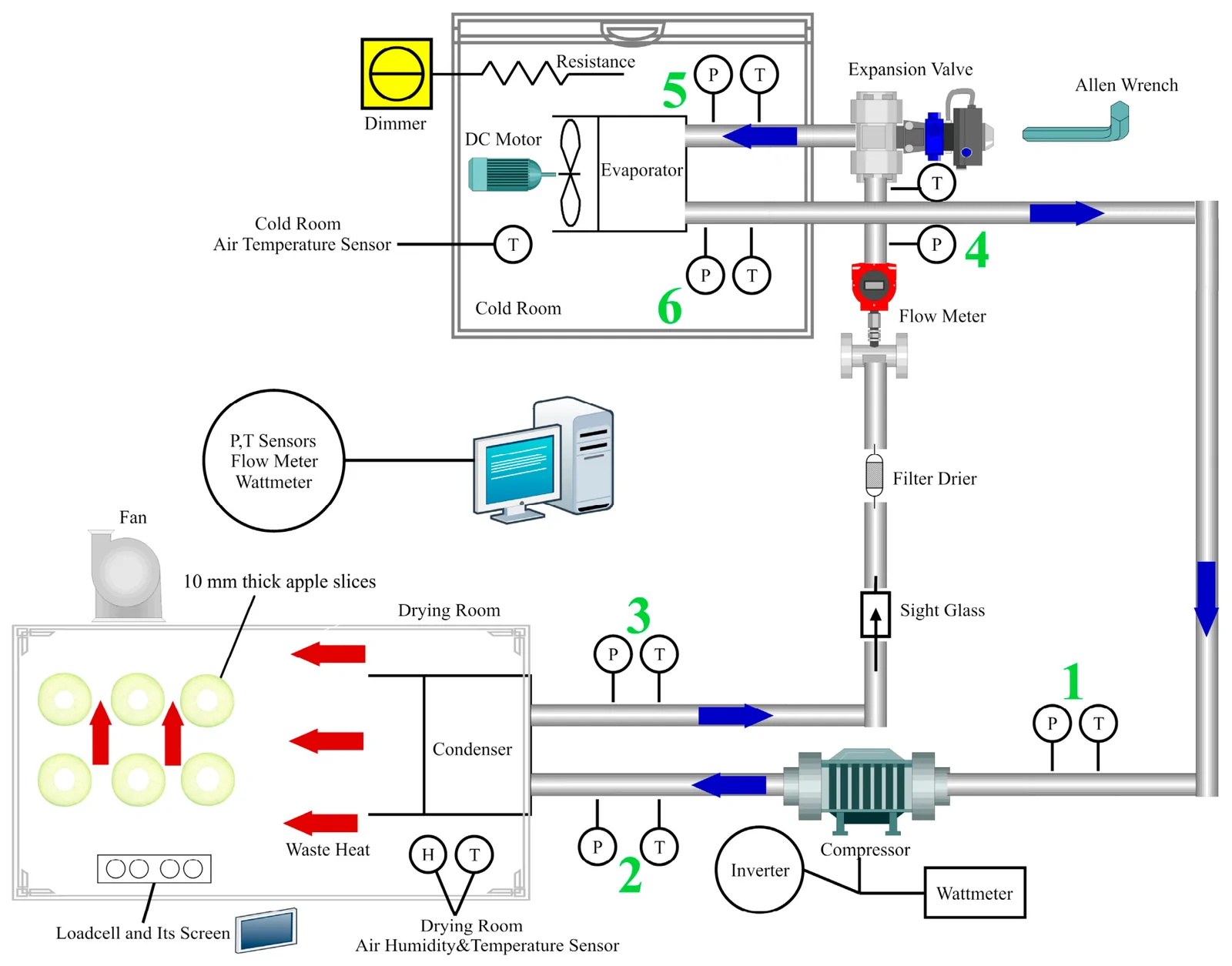
Schematic representation of refrigeration test bench.

The test bench where the performance of R290 and R600a refrigerants was compared experimentally is shown schematically in Figure 2. The main components of the refrigeration test bench are an evaporator, a hermetic compressor, a condenser, and an expansion valve. The sub-components of the experimental setup are a flow meter, a filter drier, a sight glass, pressure sensors, temperature sensors, a wattmeter to measure compressor power consumption, and a vacuum pump utilized to avoid any moisture and unwanted air during refrigerant replacement. The flow rate of R290 and R600a circulating in the system was determined using a turbine-type flowmeter. Then, the volume flow rate of these refrigerants measured by the turbine flowmeter was converted into mass flow rate by multiplying by density. In the experimental studies available in the literature on refrigeration systems, researchers generally made calculations based on mass flow rate [26,27,28,29]. In this way, it was aimed to provide unit consistency with the literature in terms of flow rate and to make a meaningful comparison of the results obtained. Table 2 includes the technical details of measurement instruments in the test bench.

In this study, the control strategy for evaporator and condenser temperatures included using many components mounted to the refrigeration test bench. The first significant temperature drop in the evaporator temperature was achieved with rotation of the screw located inside the expansion valve. Then, minor adjustments in the evaporator temperature were made by changing the speed of the evaporator fan and, at the same time, providing a thermal load to the cold room with the electrical resistance. While these adjustments are made in the evaporator temperature, it is inevitable that there will be changes in the condenser temperature. For example, while the evaporator temperature is set to −16 °C and at the same time the condenser temperature is desired to be 30 °C, the condenser temperature in the system can be 28 °C or 32 °C. At this point, the system was intervened by changing the rotational speed of the condenser fan, and thus the condenser temperature was adjusted to the desired value. That is, when it is desired to reach a high refrigerant temperature of 40 °C in the condenser, the condenser fan was operated at around 30–35% using the PLC screen. The purpose of reducing the rotational speed of the condenser fan was to reduce the amount of heat convection occurring between the air and refrigerant by reducing the amount of air passing through the condenser. On the contrary, when condenser temperatures as low as 25 °C were aimed for, the fan of the condenser was set to 100% by means of the PLC screen, and effective cooling was provided in the condenser.

Operational conditions utilized in this study, which were identical for both refrigerants, are given in Table 3. The evaporator temperature was kept constant at −16 °C, which lies within the range of medium-temperature refrigeration applications (−25 °C < T_evap_ < 0 °C) [30]. At a given condenser temperature, a lower evaporator temperature increases the compression ratio and, consequently, the compressor discharge temperature [27,30]. Since the compressor outlet is directly connected to the condenser inlet, this provides superheated vapor at a higher temperature at the condenser inlet, which is favorable for heating the drying air. The condenser temperature was varied between 25 and 40 °C to represent different ambient temperature conditions [27] and to investigate its effect on the refrigeration performance and the condenser waste heat, since it directly determines the temperature level at which the waste heat is transferred to the drying air.

Experiments were performed three times for all refrigerants. The experiments were performed under fixed conditions for 25–35 min. After stable conditions were reached, the necessary data for the thermodynamic analysis were selected. Changes in condenser and evaporator temperatures within a ±0.2 °C interval were accepted for steady-state conditions. In addition, the degree of subcooling varied according to the operating conditions. The highest alteration in the superheating degree was determined as ±1 °C.

### 2.2. Equations for Thermodynamic Analysis

Equations (1)–(4) are the most fundamental equations used in refrigeration/heat pump applications [31].

The cooling capacity is calculated with Equation (1).

(1) $${\dot{Q}}_{\mathrm{evap}}={\dot{m}}_{\mathrm{measured}}(h_{\mathrm{evap},o}-h_{\mathrm{evap},i})$$

COP of refrigeration system is calculated with Equation (2).

(2) $${\mathrm{COP}}_{R}=\frac{{\dot{Q}}_{\mathrm{evap}}}{{\dot{W}}_{\mathrm{comp}}}$$

Heat pump COP of the system is calculated with Equation (3)

(3) $${\mathrm{COP}}_{\mathrm{HP}}=\frac{{\dot{Q}}_{\mathrm{cond}}}{{\dot{W}}_{\mathrm{comp}}}$$

Heat rejected from the condenser is calculated with Equation (4).

(4) $${\dot{Q}}_{\mathrm{cond}}={\dot{m}}_{\mathrm{measured}}(h_{\mathrm{cond},i}-h_{\mathrm{cond},o})$$

### 2.3. Uncertainty Analysis

The total error can be calculated with the help of Equation (5), which is based on the root-sum-square error propagation method [32]. In this equation, the total uncertainty is represented by W_R_, the measured parameter is symbolized by R, the uncertainties caused by the experimental measurement errors in the tests are shown by x_1_, x_2_, …, x_n_, and the error rates associated with the independent variable are indicated by w_1_, w_2_, …, w_n_. Table 4 presents uncertainty values that may be encountered in the measurements.

(5) $$W_{R}={\left[{\left(\frac{\delta R}{{\delta x}_{1}}w_{1}\right)}^{2}+{\left(\frac{\delta R}{{\delta x}_{2}}w_{2}\right)}^{2}+..........+{\left(\frac{\Delta R}{{\Delta x}_{n}}w_{n}\right)}^{2}\right]}^{1/2}$$

### 2.4. Drying Process

In this study, the drying performance of two different natural refrigerants, R290 and R600a, was comparatively investigated under the respective operating conditions. During the drying experiments, the product mass was recorded at one-minute intervals, and the moisture content, moisture ratio, and drying rate were calculated for each time step. In the calculations, the final dry mass was assumed to be 34 g. Accordingly, both wet-basis and dry-basis moisture contents were determined from the change in product mass.

The drying experiments were conducted with a measurement interval of 1 min. The R290 experiment lasted 265 min, whereas the R600a experiment lasted 300 min. The average drying temperature was taken as 313 K for R290 and 300 K for R600a. For the energy performance calculations, the total energy consumption was taken as 1.850 kWh for R290 and 1.000 kWh for R600a. The half-thickness of the product was assumed to be 5 mm for the calculation of the effective moisture diffusivity. The corresponding parameters are presented in Table 5.

#### Calculations of Moisture Content, Moisture Ratio, and Drying Rate

The drying kinetics were evaluated based on the time-dependent mass loss of the product. For this purpose, the product mass at any given time (t) was denoted as (W_t_), while the dry matter mass and initial product mass were denoted as (W_d_) and (W_0_), respectively. The wet-basis moisture content represents the amount of water relative to the total product mass, whereas the dry-basis moisture content was calculated as the ratio of the water mass to the dry matter mass. In thin-layer drying studies, the moisture ratio and drying rate are widely used to model drying curves and compare different drying conditions [33,34,35].

(6) MC_wet_ = ((W_t_ − W_d_)/W_t_) × 100

(7) MC_dry_ = (W_t_ − W_d_)/W_d_

(8) MR = (M_t_ − M_e_)/(M_0_ − M_e_)

Since the equilibrium moisture content (M_e_) is considered sufficiently low compared with the initial and instantaneous moisture contents under the experimental conditions, the moisture ratio was simplified as follows:

(9) MR ≈ M_t_/M_0_

(10) DR = (M_t_ − M_t+Δt_)/Δt

In this context, MC_wet_ denotes the wet-basis moisture content (%), MC_dry_ denotes the dry-basis moisture content (g water/g dry matter), MR denotes the dimensionless moisture ratio, DR denotes the drying rate, M_t_ denotes the dry-basis moisture content at time t, M_0_ denotes the initial dry-basis moisture content, and Δt denotes the measurement time interval. In this study, Δt = 1 min was used.

### 2.5. Artificial Intelligence Modeling Process

**Figure 3:**
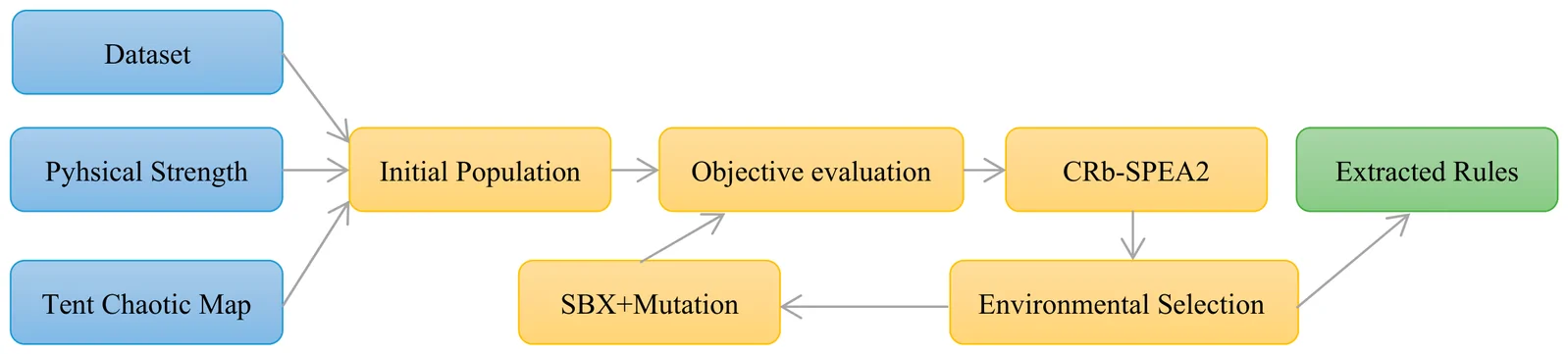
P-CRb-SPEA2 general structure.

In this study, an enhanced version of the CRb-SPEA2 algorithm, previously developed by the authors, namely the Physics-Guided Ordinal CRb-SPEA2 (P-CRb-SPEA2) algorithm, is proposed [36]. The proposed method extracts interpretable classification rules for problems involving ordinal class structures. The method extends the fitness assignment and archive management strategies of SPEA2 by incorporating four additional components. These components include an ordinal error objective that directly optimizes the ordering of classes, a physical consistency objective that incorporates domain knowledge into the objective function, a complexity objective to ensure interpretability, and a chaotic initialization component to provide a more uniformly distributed search space. In addition to these components, a null-rule repair mechanism is incorporated to eliminate invalid solutions that may arise in the rule-based representation. The output of the proposed method is not a single model; rather, it is a Pareto rule set that provides the decision-maker with trade-offs among coverage, ordinal reliability, physical consistency, and simplicity. The methodology followed in this study consists of the formal definition of the problem, representation of candidate solutions, formulation of four objective functions for many-objective optimization, development of the components of the search algorithm, and subsequent rule extraction and evaluation of the relevant performance metrics. The overall workflow of the proposed method is presented in Figure 3.

#### 2.5.1. CRb-SPEA2 Algorithm

The SPEA2 algorithm is a Pareto-based elitist evolutionary algorithm developed for solving multi-objective and many-objective optimization problems. Designed to overcome the limitations of the original SPEA algorithm in terms of its fitness assignment mechanism and archive management, SPEA2 preserves Pareto dominance within the search space. At the core of SPEA2 is a fitness assignment mechanism that combines the principles of dominance strength and density estimation. For each individual i in the population (Pt) and archive ($\bar{P_{t}}$), a Strength value, S(i), representing the number of solutions dominated by that individual, is calculated. Subsequently, the raw fitness value, R(i), is obtained as the sum of the strength values of all solutions j that dominate individual i (Equation (11)) [37].

(11) $$R\left(i\right)=\sum_{j\in P_{t}\cup \bar{P_{t}},j≻i}S(j)$$

To prevent excessive clustering in the solution space and distinguish individuals with the same raw fitness value, a density metric based on the k-nearest neighbor method is incorporated into the fitness function (Equation (12)) [37].

(12) F(i) = R(i) + D(i)

Elitism is implemented through a fixed-capacity external archive ($\bar{N}$) defined in advance. During the environmental selection stage, all nondominated solutions satisfying F(i) < 1 are transferred to the new archive. If the number of individuals in the archive does not exceed the predefined archive size, the best solutions from the population are added. If the archive size is exceeded, a specific truncation operator is activated. During truncation, individuals with the shortest neighborhood distances in the objective space are sequentially removed, while boundary solutions are preserved. In this way, both convergence toward and diversity along the Pareto front are maintained.

In the CRb-SPEA2 method, the Tent chaotic map is incorporated into SPEA2 to ensure a more uniform distribution of candidate solutions throughout the search space and to prevent premature convergence (Equation (13)) [38]. The algorithm has demonstrated successful performance, particularly in explainable artificial intelligence and rule-based multi-/many-objective classification problems. While conventional machine learning algorithms generally provide a single solution to a given problem, this algorithm generates rule sets that explain complex relationships within the dataset. In the algorithm, the representation of candidate solutions is first defined, followed by the determination of how these solutions will be evaluated during rule extraction. In the CRb-SPEA2 algorithm, candidate solutions are represented using three different vectors [36]. For the Ci candidate solution, the vectors $\vec{C_{i}^{b}}$, $\vec{C_{i}^{l}}$, and $\vec{C_{i}^{u}}$ indicate, respectively, whether the corresponding attribute is included in the rule and the lower and upper limit values that the attribute can take. The ability to determine which attributes are included in the rule and to optimize their corresponding values without discretization constitutes an important contribution of the method. In Equation (14), ci values represent the lower and upper bounds of Fi attributes included in the rule, while S denotes the class to which the rule belongs.

(13) $$X_{n+1}=\left\{\begin{array}{cc} \alpha X_{n}, & X_{n}<0.5\\ \alpha \left(1-X_{n}\right),\; & X_{n}\geq 0.5 \end{array}\;(\alpha =1.99)\right.$$

(14) $$\mathrm{if}\;c_{3}^{l}\leq F_{3}\leq c_{3}^{u}\;\mathrm{and}\;c_{5}^{l}\leq F_{5}\leq c_{5}^{u}\;\mathrm{and}\;c_{7}^{l}\leq F_{7}\leq c_{7}^{u}\;\mathrm{then}\;S$$

#### 2.5.2. P-CRb-SPEA2 Algorithm

The P-CRb-SPEA2 version of the CRb-SPEA2 method, specifically developed for this study, is a physics-guided multi-objective metaheuristic method capable of extracting interpretable rules for problems with an ordinal class structure. In addition to the CRb-SPEA2 framework, the proposed method incorporates an ordinal error objective, a physical consistency objective, and a complexity objective.

For a training dataset with n samples and d continuous attributes *D* = {(*x_i_*, *y_i_*)} (*i* = 1, 2, …, *n*), let the attribute vector be *x_i_* ∈ ℝ*^d^*, and the corresponding class label be *y_i_* = {*C*_1_, *C*_2_, …, *C_q_*}. Unlike conventional classification approaches, the classification structure in the proposed algorithm is based on an ordered set of classes (Equation (15)).

(15) *C*_1_ ≺ *C*_2_ ≺, …, ≺ *C_q_*

This ordering indicates that the distance between classes is important in terms of classification accuracy. In other words, predicting class C_3_ instead of class C_1_ constitutes a more unacceptable error than predicting class C_2_. This ordinal structure can be encountered in real-world problems, particularly in process monitoring, risk assessment, and maturity classification. The structure used for rule extraction is presented in Equation (16).

(16) *IF* (*l_j_* ≤ *x_j_* ≤ *u_j_*) *THEN y* = *C_t_*

**Figure 4:**
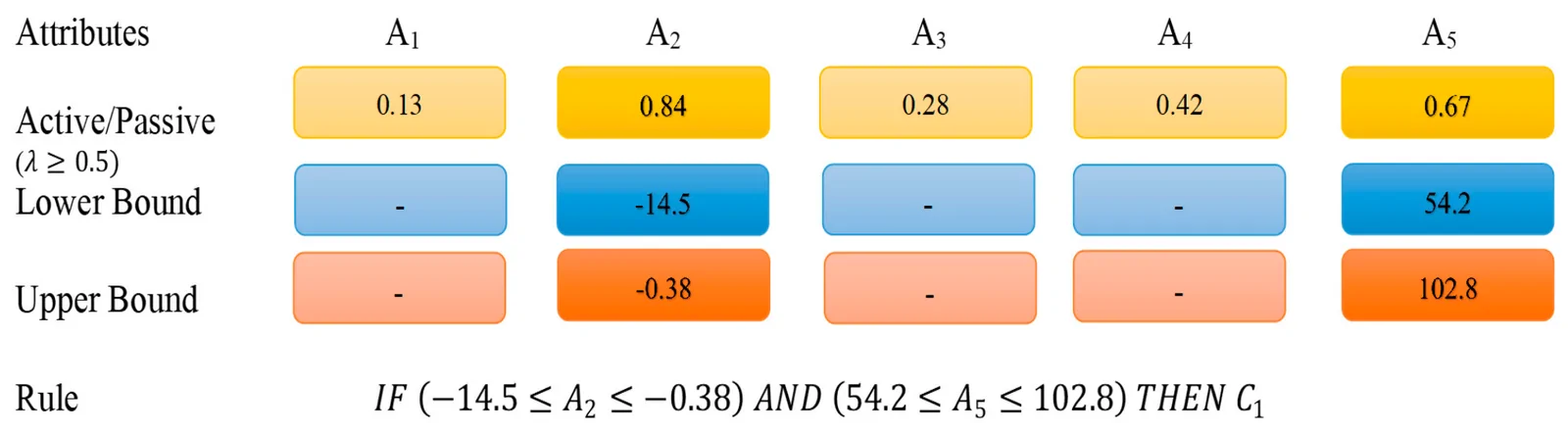
Candidate solution representation and its transformation into a rule.

Here, x_j_ denotes the j-th attribute, while l_j_ and u_j_ represent the lower and upper bounds of the interval associated with the corresponding attribute, respectively. Learning is performed directly on the boundaries of these intervals, which consist of continuous values, and attribute selection is carried out as part of the optimization process rather than as a separate preprocessing step. Each candidate solution vi is represented by three real-valued sub-vectors. The first of these vectors determines whether the corresponding attribute is included in the rule. In this study, this condition is determined using a threshold value λ = 0.5. The lower and upper bounds of the attributes are denoted by l = [l_1_, l_2_, …, l_d_] and u = [u_1_, u_2_, …, u_d_], respectively (l_j_, u_j_ ∈ R). During rule decoding, only active attributes (i.e., those exceeding the threshold value) are considered, whereas the lower and upper bounds of inactive attributes are retained but are not included in the rule. Thus, an attribute can be temporarily deactivated and subsequently reactivated during the search process while preserving its previously learned interval. An example of the transformation of attribute values into rules is presented in Figure 4.

In this study, the quality of the candidate solutions was evaluated based on Recall, Ordinal Error, Physical Consistency Index, and Rule Complexity. In the proposed method, a different measure was employed for each objective, namely the confusion matrix, error order, interval position, and number of conditions. In the confusion matrix used for the evaluation, the “if” and “then” parts of a rule were evaluated separately when comparing a rule with a given data instance (Table 6).

Recall is a performance metric that indicates the percentage of all actual positive instances that are correctly identified by a model (Equation (17)).

(17) Recall = True Positive/(True Positive + False Negative)

Since the classes in the problem considered in this study are ordinal, the severity of a misclassification is not necessarily the same for all classes. Assigning an instance to a class that is farther away in the ordinal sequence constitutes a more severe error than assigning it to an adjacent class. Since conventional performance metrics cannot account for this distinction, ordinal error was selected as one of the performance metrics. Let t denote the target class index and let the predicted class be C_t_. During rule evaluation, each instance whose actual class is y = C_k_ (k ≠ t) produces a penalty score proportional to its distance from the target class in the ordinal sequence, and the sum of these penalties is referred to as the ordinal false positive (OFP) (Equations (18)–(20)). In the equations, n denotes the number of instances in the training dataset, x denotes the attribute vector, y denotes the actual class, q denotes the total number of classes, C_t_ denotes the target class of the rule, β(x_i_) denotes the rule body indicator (0/1), κ(i) denotes the ordinal class index of the i-th instance, n_t_–denotes the number of instances that do not belong to the target class, and OrdErr denotes the normalized ordinal error.

(18) $$\mathrm{OFP}=\sum_{i=1}^{n}\beta \left(x_{i}\right)\;\left|\kappa \left(i\right)-t\right|$$

(19) $$n_{t^{-}}=\left|\left\{i:y_{i}\neq C_{t}\right\}\right|=\mathrm{TN}+\mathrm{FP}$$

(20) $$\mathrm{OrdErr}=\frac{\mathrm{OFP}}{n_{t^{-}}}=\frac{1}{n_{t^{-}}}\sum_{i=1}^{n}\beta \left(x_{i}\right)\left|\kappa \left(i\right)-t\right|,\mathrm{OrdErr}\in \left[0,\;q-1\right]$$

A statistically discriminative attribute does not necessarily have a causal relationship with the training process. An attribute that varies within a narrow range and at a level comparable to the measurement noise may appear to be discriminative when its within-class variability is low. Consequently, rules containing such attributes may provide high accuracy but may be difficult to justify from a physical or domain-knowledge perspective. The Physical Consistency Index (PCI), one of the objectives of the proposed method, is employed to address this issue. For each attribute j in the dataset, a physical strength value (g_j_ ∈ [0, 1]) is calculated. In Equations (21)–(24), $\mu_{j}^{\left(k\right)}$ denotes the mean value of attribute j in class C_k_, while S_j_ denotes the standard deviation of the attribute in the corresponding dataset. Δ_j_ represents the total magnitude of variation exhibited by attribute j throughout the drying process, and A denotes the active attributes. The monotonicity factor, denoted by ρ_j_, determines whether the class means change monotonically from C_1_ to C_q_. If a process progresses in a single direction, an attribute whose values change monotonically across the class sequence is considered physically consistent.

(21) $$g_{j}=\frac{\mathrm{std}(\mu_{j}^{\left(1\right)},\;\ldots ,\mu_{j}^{\left(q\right)})}{S_{j}}\times \rho_{j}\times \min(1,\frac{\Delta_{j}}{\Delta_{\min}})$$

(22) $$\rho_{j}=\left\{\begin{array}{c} 1,{\;\mu }_{j}^{\left(1\right)}<\ldots <\mu_{j}^{\left(q\right)}\;\mathrm{or}\;\mu_{j}^{\left(1\right)}>\ldots >\mu_{j}^{\left(q\right)}\\ 0.3,\;\mathrm{elsewhere} \end{array}\right.$$

(23) $$\Delta_{j}=\left|\mu_{j}^{\left(q\right)}-\mu_{j}^{\left(1\right)}\right|$$

(24) $$\mathrm{PCI}=\frac{1}{\left|A\right|}\sum_{j\in A}g_{j}\in [0,\;1]$$

The other objective, rule complexity, is defined as the number of active attributes used in the rule (Equation (25)). This objective aims to optimize the interpretability of the rule. In addition, single-condition and multi-condition alternative solutions are presented side by side on the same Pareto front, providing the decision-maker with alternatives in terms of simplicity and coverage.

(25) *Complexity* = |*A*|, (*a_j_* ≥ *λ*)

When all objectives are considered together, the objective vector of the i-th candidate solution is expressed as given in Equation (26). Since the objectives are defined on different scales, min–max normalization was applied to the current population before density estimation.

(26) *O_i_* = [*Recall*, *OrdErr*, *PCI*, *Complexity*]

In addition to all these operations, if the activation value of a candidate falls below the threshold (max_j_ a_j_ < λ), the rule body becomes empty. Since an empty rule body would match every instance, the number of false negatives becomes zero, and the Recall value is consequently equal to 1. Such a candidate solution can never be dominated with respect to the Recall objective and therefore remains permanently on the Pareto front, continuously occupying a position in the archive. To prevent this undesirable situation, a repair operator was introduced, which reactivates the attribute with the highest activation value using a chaotic value when none of the attributes is active. The operator is executed both during population initialization and after each genetic operation. No empty rules were encountered throughout all iterations of the experiments. The pseudocode of the P-CRb-SPEA2 algorithm is presented in Algorithm 1.
**Algorithm 1.** Pseudo code of P-CRb-SPEA2Input: D     : training set                  Output: Pareto set of non-dominated rules            *C_t_*    : target class            P     : population            H    : mating pool            Q    : offspring            λ     : active/passive threshold            N    : population size            N^−^   : archive size            T     : max iterations            *α*     : chaotic parameter            *p_c_*    : crossover probability              1.      Assign class order *C*_1_, *C*_2_, …, *C_k_*, *rank*(*C_k_*) = *k*             2.      Compute physical strength *g_j_* for every attribute *j* from *D*             3.      Initialize tent chaotic map with (*X*_0_, *α*)             4.      P ← ∅             5.      for i = 1 to N do             6.                 generate vectors *a*, *l*, *u* of *V_i_* using the chaotic map             7.                 BoundRepair(*V_i_*)             8.                 RuleRepair(*V_i_*)             9.                 *P* ← *P* ∪ {*V_i_*}           10.     end for           11.     Archive ← ∅           12.     Evaluate(P, D)           13.     Normalize objectives and compute Strength, Raw, Density, Fitness over P           14.     Archive ← EnvironmentalSelection(P)           15.     for it = 1 to T do           16.     H ← binary-tournament mating pool from *P* ∪ *Arc*, |*H*| = *N*/2           17.                Q ← ∅           18.     for h = 1 to |H| do           19.                 if chaotic() < *p_c_* then           20.                                *Q* ← *Q* ∪ *SBX*(*H*)           21.                 else           22.                              *Q* ← *Q* ∪ *PolynomialMutation*(*H*)           23.                 end if           24.     end for           25.     for each *V* ∈ *Q* do BoundRepair(V); RuleRepair(V) end for           26.     Evaluate(Q, D)           27.     *M* ← *P* ∪ *Q* ∪ *Archive*           28.     Normalize objectives over M           29.     Compute Strength, Raw, Density, Fitness over M           30.     Archive ← EnvironmentalSelection(M)           31.     P ← new population drawn from Archive           32.     iteration ← iteration + 1           33.     end for           34.     Return non-dominated members of Archive

#### 2.5.3. Application of the Proposed Method to the Problem

The proposed method was applied to determine the drying stage in a heat pump dryer. Continuous weighing of the product during industrial applications is not a practical method. Therefore, in this study, stage identification was planned based solely on the temperature signature of the cycle, without any physical contact with the product being processed. The dataset obtained from experiments conducted using two different refrigerants, R290 and R600a, was used in the analysis (Table 7). The moisture ratio was categorized into three ordinal stages as presented in Table 8.

**Figure 5:**
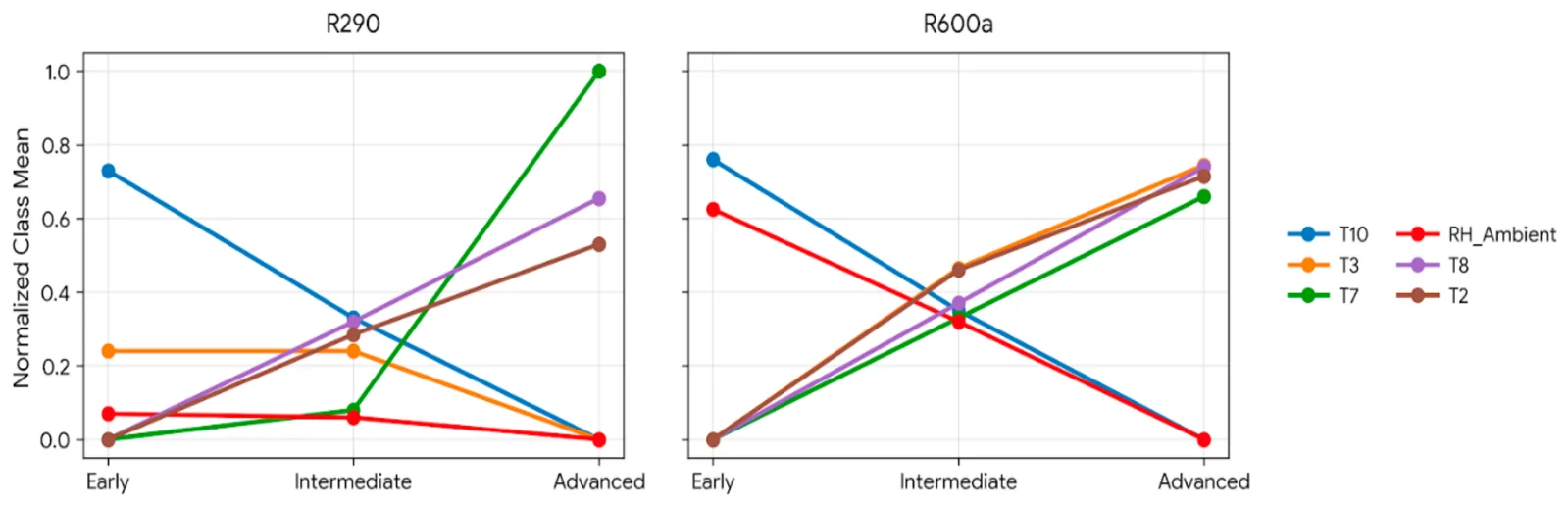
Normalized class averages of attributes throughout drying stages.

The applicability of the PCI objective to the problem considered in this study depends on whether the attributes exhibit genuinely monotonic changes across the drying stages. Figure 5. presents the class means of each attribute normalized according to their respective ranges. For R600a, all attributes exhibit monotonic changes throughout the stages. For R290, the T_10_ and T_8_ attributes show distinct trends, whereas the T3 and RH_Ambient_ attributes remain nearly constant.

In the experimental studies, the population size and archive capacity were both set to 20, while the number of iterations was set to 50, and 30 independent runs were performed. The distribution indices for SBX and polynomial mutation were set to 5, with a crossover probability of 0.9, a parent pool size of 10, and an activation/deactivation threshold of 0.5. Independent optimizations were conducted for a total of six distinct “refrigerant, stage” pairs. The performance of the resulting models was compared with conventional machine learning methods, namely Naive Bayes, k-NN (k = 5), SVM, Decision Tree, and Random Forest, using unscaled data. The quality of the Pareto front was evaluated using the Hypervolume (HV), IGD, and Spacing metrics with respect to a reference front constructed from the combined nondominated set of all runs. To assess the dependence of the results on a single data split, the entire process was conducted using 10-fold cross-validation, and the variability across folds was reported in terms of standard deviation values.

## 3. Results and Discussion

In this section, the refrigeration performance of R290 and R600a is first presented and discussed, followed by the drying performance findings obtained using condenser waste heat. Finally, the results of the proposed P-CRb-SPEA2 algorithm for drying-stage identification are presented and compared with classical machine-learning models.

### 3.1. Refrigeration Performance Findings of R290 and R600a

**Figure 6:**
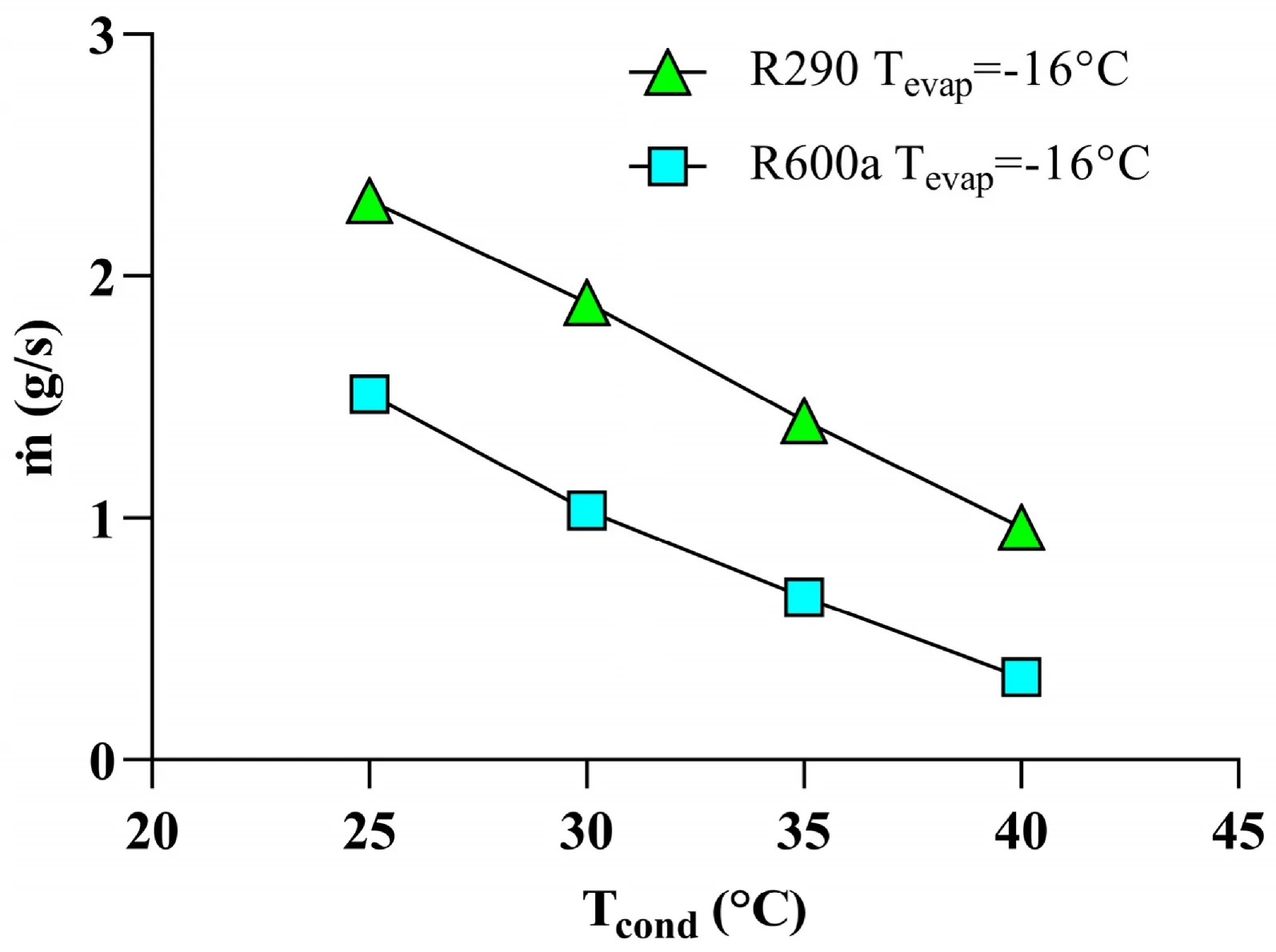
Mass flow rate variation in utilized refrigerants at different experimental conditions.

Figure 6 shows the effect of different operational temperatures on the mass flow rate of the refrigeration system. It is seen from the figure that a lower condenser temperature causes a higher refrigerant mass flow rate at a certain evaporation temperature. For example, the mass flow rate of R290 at an evaporator temperature of −16 °C was 2.31, 1.89, 1.40, and 0.96 g s^−1^ for condenser temperatures of 25 °C, 30 °C, 35 °C, and 40 °C, respectively. The mass flow rate of R290 refrigerant showed higher values than R600a under all operational conditions. The average mass flow rate measurements were 1.64 g s^−1^ for R290 and 0.89 g s^−1^ for R600a. In brief, the average mass flow rate of R290 was almost 1.84 times that of R600a. The higher mass flow rate of R290 was caused by its greater vapor density than R600a.

**Figure 7:**
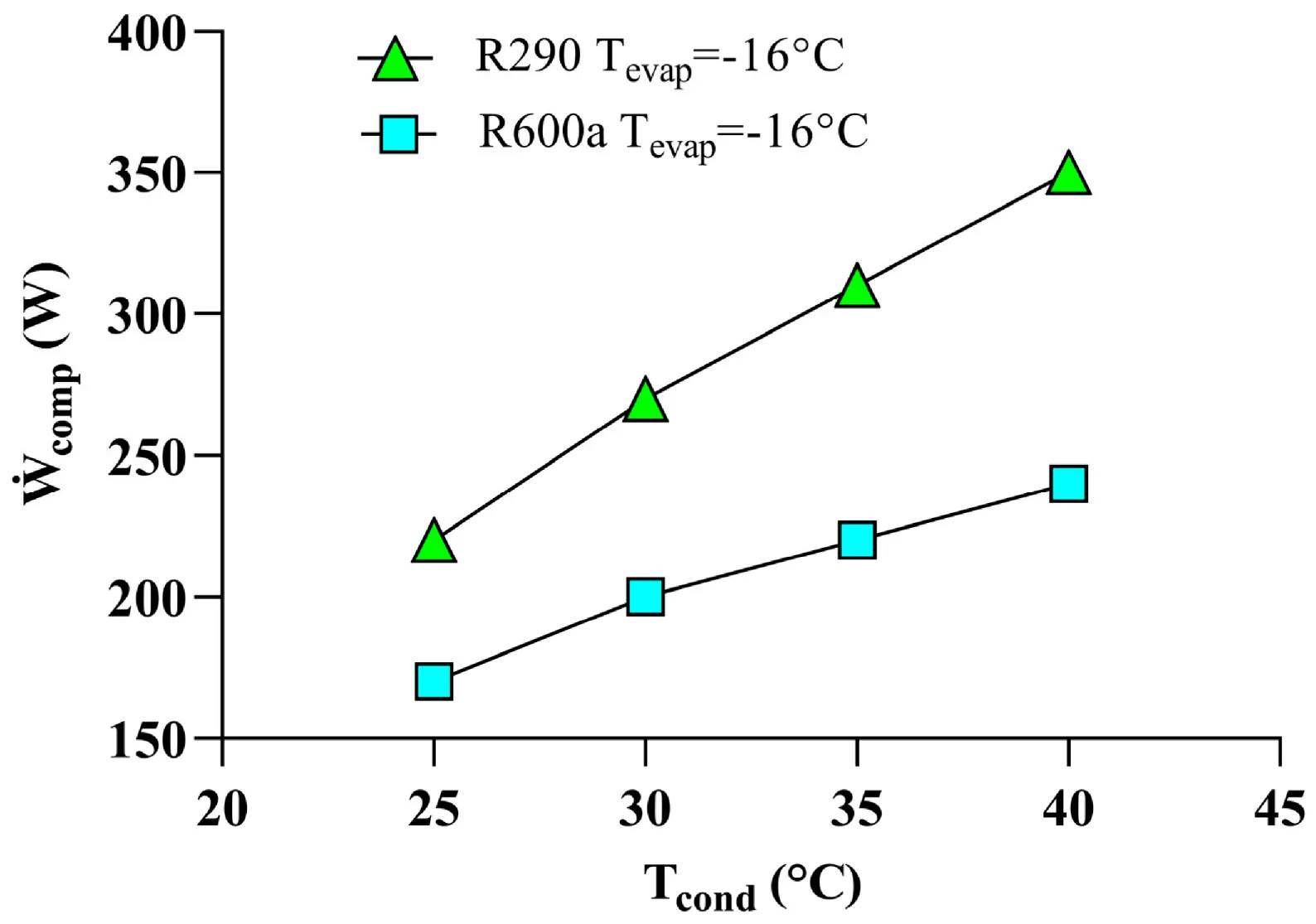
Change in compressor power under varying experimental conditions.

Figure 7 indicates the change in power consumption at different operational temperatures. At a constant evaporator temperature of −16 °C, increasing the condenser temperature from 25 °C to 40 °C results in an increase in compressor power consumption for both refrigerants, with the increase being approximately 59% for R290 and 41% for R600a. This difference can be attributed to the distinct thermophysical properties and operating pressure levels of the refrigerants. Compressor power consumption values of R290 were detected to be greater than R600a in all experimental operational conditions. The average of $\dot{W}$_comp_ measurements in all operating conditions was 288 W for R290 and 208 W for R600a. Thus, R290 consumed 38.5% more power than R600a. The significantly higher critical temperature of R600a compared to R290 is the reason for the lower power consumption with R600a. R290 refrigerant consumed more power than R600a in experimental refrigeration test benches in previous works too [10,39].

**Figure 8:**
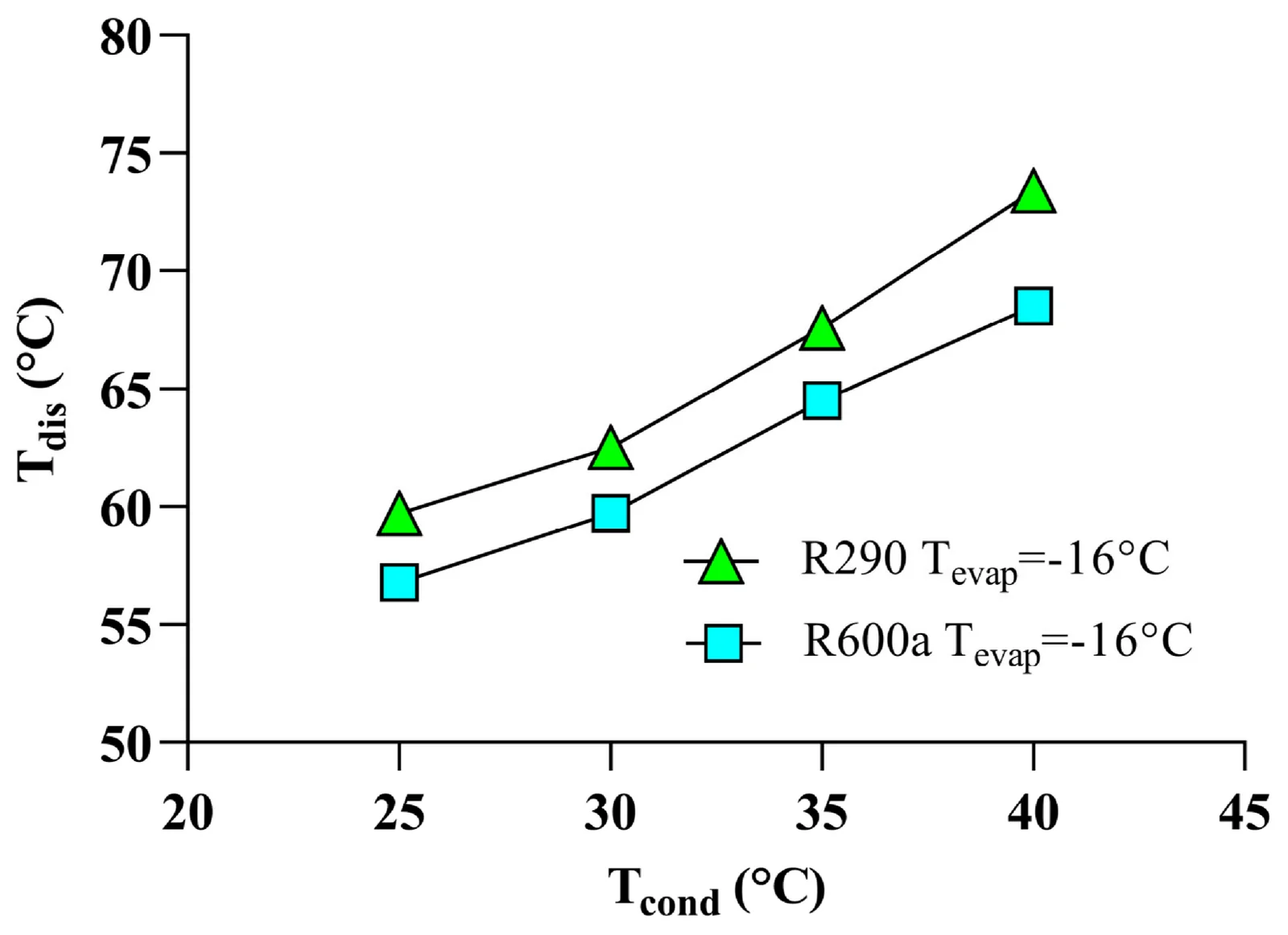
Compressor discharge temperature changes for various experimental parameters.

Figure 8 represents the behavior of compressor discharge temperature at different experimental parameters. The results indicate that, at a constant evaporator temperature of −16 °C, increasing the condenser temperature from 25 °C to 40 °C leads to an increase in T_dis_ for both refrigerants, rising from 59.7 °C to 73.4 °C for R290 and from 56.8 °C to 68.5 °C for R600a. R290 refrigerant caused greater compressor discharge temperatures than R600a in all operating parameters in the refrigeration system. The average of all measured T_dis_ values for R290 was 65.8 °C, and it was 62.4 °C for R600a. Thus, R290 showed a 5.4% increase in compressor discharge temperature compared to R600a. It is important to note that T_dis_ should be as low as possible to avoid compressor oil degradation caused by high T_dis_ values and to extend the compressor’s lifetime.

**Figure 9:**
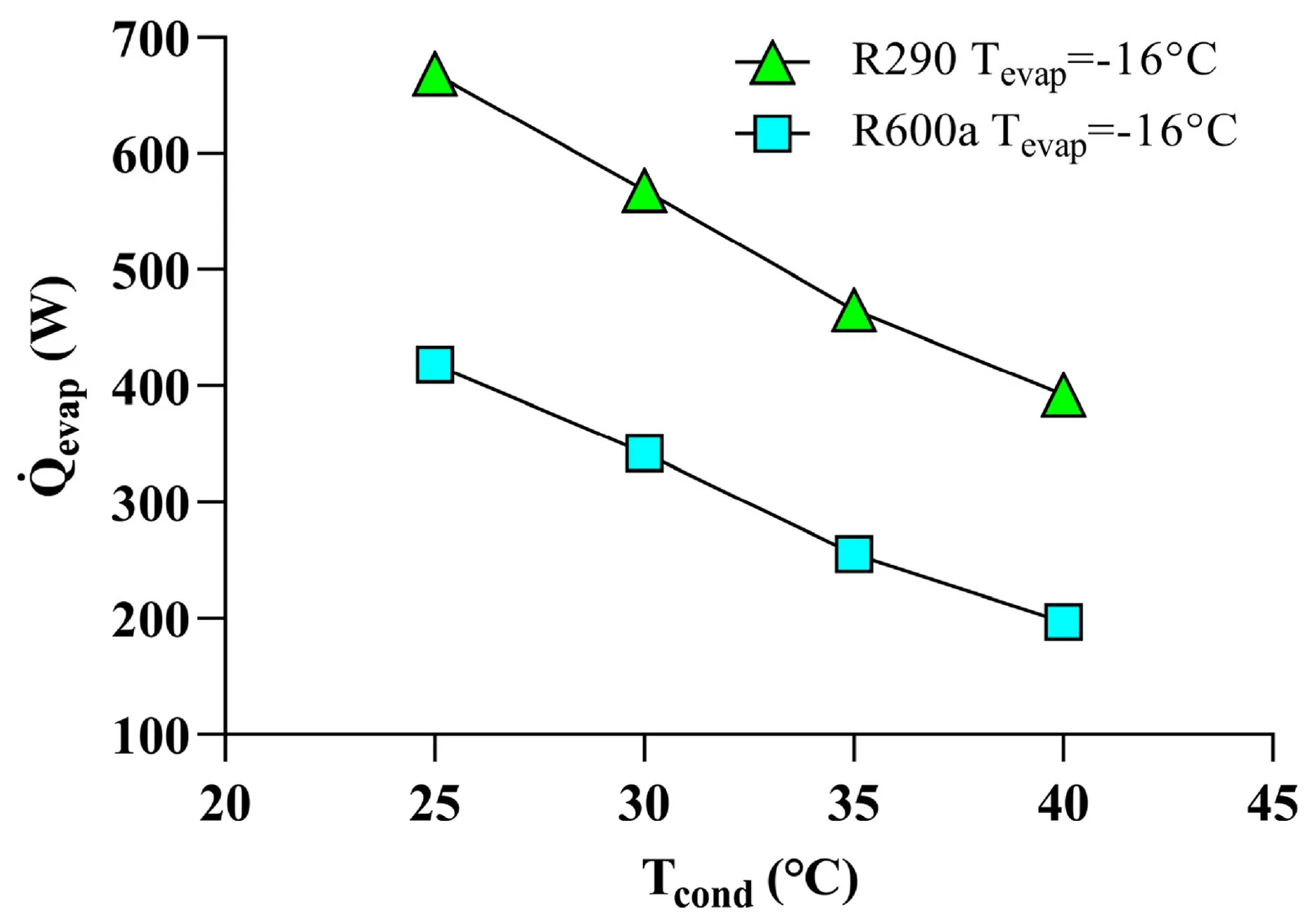
Variation in cooling capacity of the refrigeration system at different experimental parameters.

Figure 9 exhibits the alteration in cooling capacity at various experimental condensation temperatures. At a given evaporator temperature, a high condenser temperature results in lower cooling capacity values, as in the literature [29,40]. Increasing the condenser temperature from 25 °C to 40 °C results in a 41.4% decrease in the cooling capacity of R290, while the cooling capacity of R600a decreases by 52.9%. It has been stated in previous studies in the literature that for lower condensation temperatures, the cooling capacities of refrigeration systems are larger with the help of the increase in refrigerating effect and mass flow rate [30,41]. $\dot{Q}$_evap_ of the system with R290 refrigerant was found to be greater than that of R600a under all operating conditions. The average $\dot{Q}$_evap_ values were 524 W for R290 and 303 W for R600a. $\dot{Q}$_evap_ of the refrigeration system using R290 was found to be 73% higher than that of R600a. The low cooling capacity with R600a was due to the lower $\dot{m}$ and latent heat of vaporization of R600a, since the lower latent heat of vaporization caused a decrease in refrigerating effect compared to R290.

**Figure 10:**
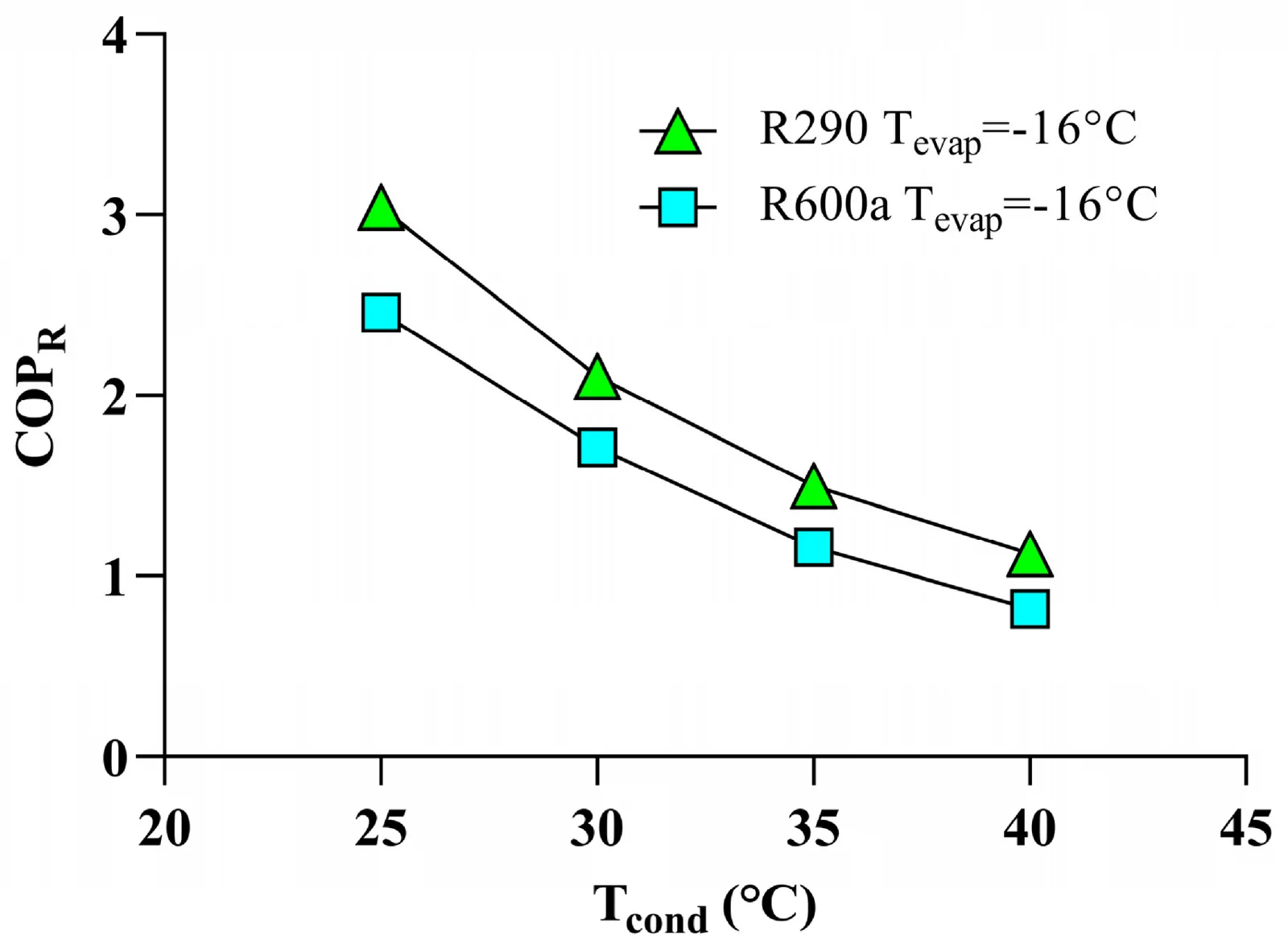
Change in COP_R_ of the refrigeration system for different parameters.

Figure 10 shows the effect of various experimental parameters on the COP_R_ of the refrigeration system. It can be observed from the figure that lower condenser temperatures resulted in higher COP_R_ values at a given evaporator temperature, as in the literature [30,40,42]. Increasing the condenser temperature from 25 °C to 40 °C leads to a reduction in the COP_R_ of both refrigerants, with the COP_R_ of R290 decreasing by 63.2% and that of R600a decreasing by 66.7%. Considering all measurement points in the refrigeration system, an average COP_R_ value of 1.94 was achieved with R290 refrigerant, while an average COP_R_ value of 1.54 was achieved with R600a. That is, the R290 refrigerant caused about a 26% increase in COP_R_ compared to R600a. As mentioned in Figure 7, R290 refrigerant caused higher compressor power consumption compared to R600a. As stated in Figure 9, R290 refrigerant caused a significant increase in cooling capacity over R600a. In the COP_R_ calculation, although R290 refrigerant seems to be disadvantageous compared to R600a due to high compressor power consumption, the much larger cooling capacity of R290 compared to R600a compensated for this situation. Thus, the COP_R_ value of the system with R290 refrigerant was higher than that of R600a.

**Figure 11:**
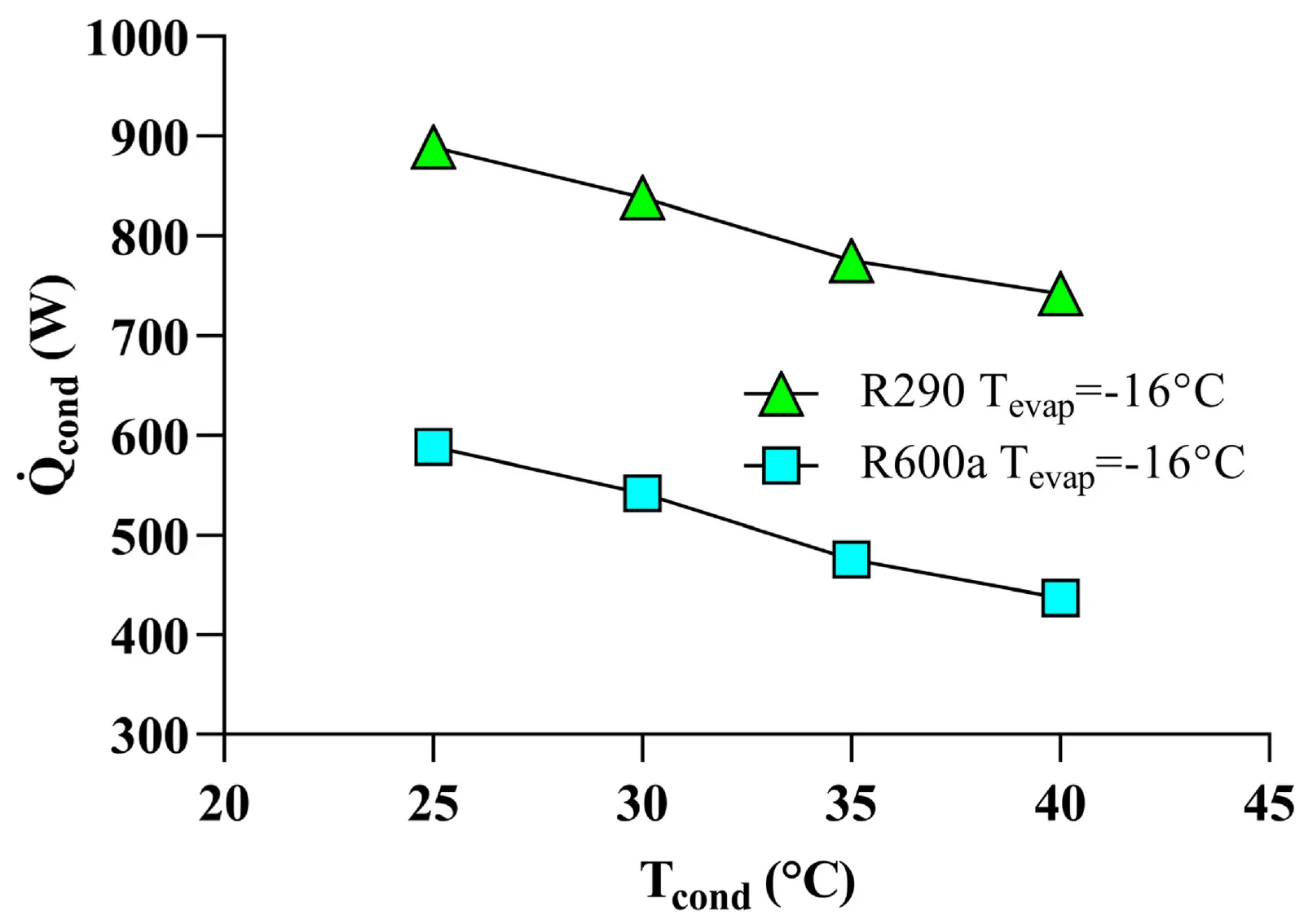
Change in condenser waste heat at varying operating temperatures.

**Figure 12:**
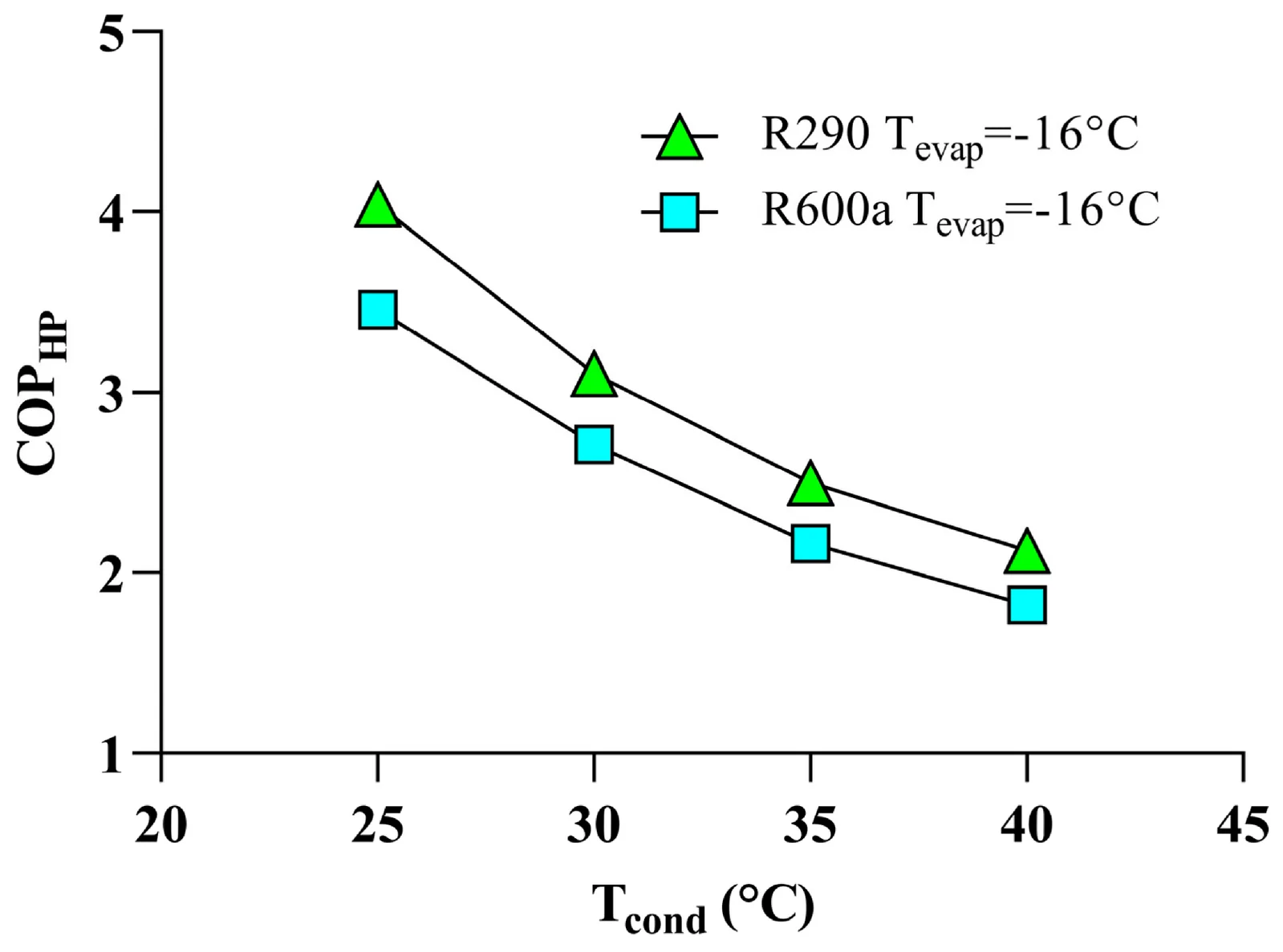
Alteration in COP_HP_ at different operating parameters.

Figure 11 and Figure 12 show the condenser waste heat and COP_HP_ findings for all operating conditions, respectively. The results indicate that the heat rejected in the condenser and COP_HP_ is higher for lower condenser temperatures at a certain evaporator temperature. Similar results were discovered in the literature [43]. When the condenser temperature decreases from 40 °C to 25 °C, the heat rejected in the condenser increases by 19.8% for R290 and 34.7% for R600a. Similarly, under the same conditions, the COP_HP_ increases by 90.6% for R290 and 90.1% for R600a. It can be seen from Figure 11 that R290 has a greater amount of waste heat through the condenser compared to R600a. When the experiments conducted at different condenser temperatures are averaged, the heat rejected in the condenser is determined to be 811 W for R290 and 510 W for R600a. The average waste heat amounts in the experiments revealed that R290 caused about a 59% increase in the $\dot{Q}$_cond_ value compared to R600a. Moreover, when the experimental COP_HP_ results presented in Figure 12 are averaged, the COP_HP_ value of R290 is found to be approximately 15.7% higher than that of R600a.

### 3.2. Drying Performance Findings of R290 and R600a

This section presents the moisture content, moisture ratio, drying rate, effective moisture diffusivity, and energy performance results obtained from the drying experiments conducted using R290 and R600a refrigerants. In the calculations, the dry matter mass of the product was assumed to be 34 g. Therefore, the amount of water removed was determined based on the difference between the initial and final product masses.

As shown in Table 9, the drying time was 265 min for the R290 experiment and 300 min for the R600a experiment. With R290, the initial product mass decreased from 170.0 g to 63.8 g, corresponding to a total water removal of 0.1062 kg. With R600a, the initial product mass decreased from 173.0 g to 63.4 g, resulting in 0.1096 kg of water removal. The initial dry-basis moisture contents for R290 and R600a were 4.000 and 4.088 g water/g dry matter, respectively, whereas these values decreased to 0.876 and 0.865 g water/g dry matter, respectively, at the end of the experiments.

**Figure 13:**
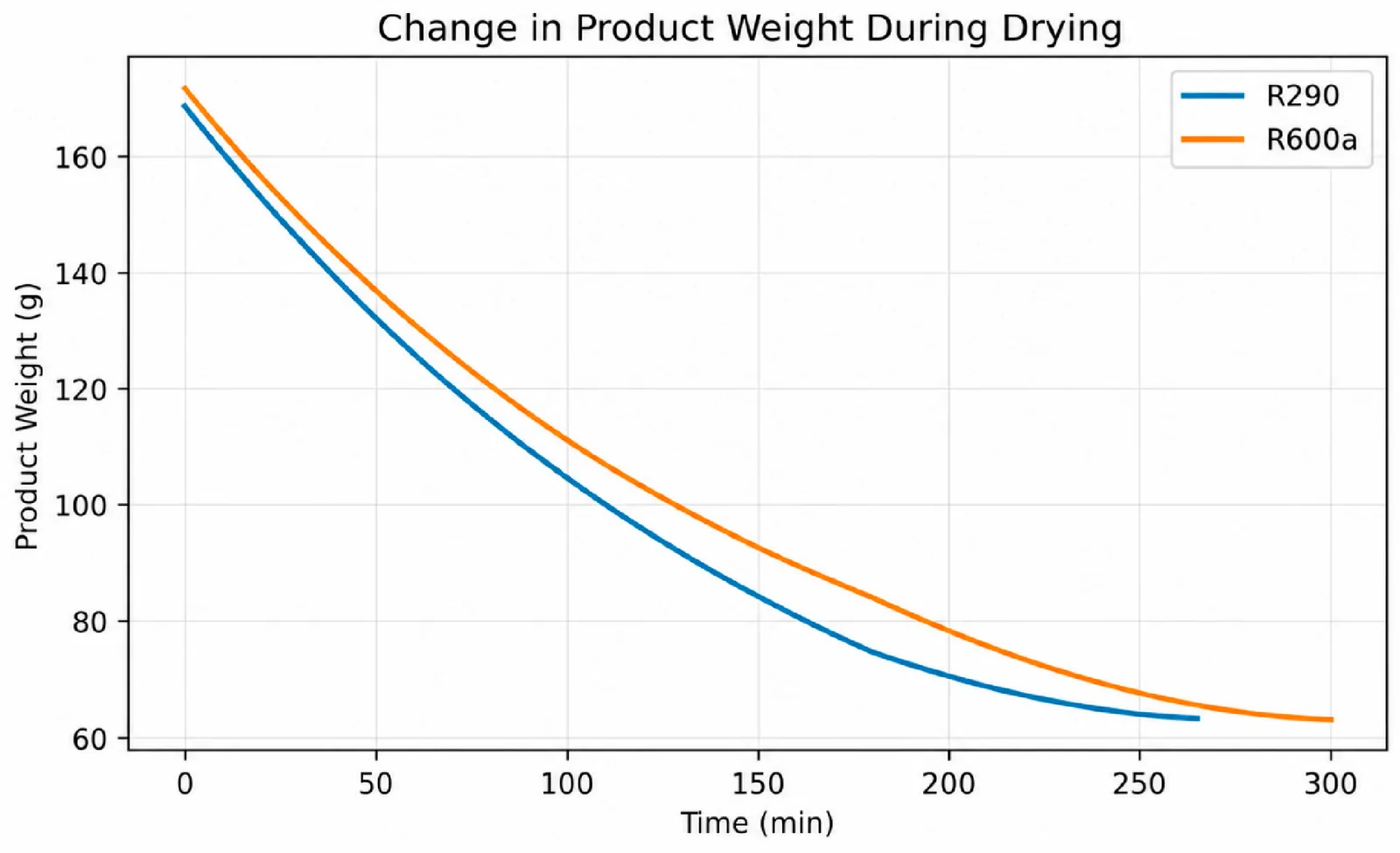
Time-dependent variation in product mass during the R290 and R600a drying processes.

Figure 13 shows that the product mass decreased steadily over time in both experiments. Under the R290 condition, the target final mass was reached in a shorter period, indicating that the drying kinetics were faster with this refrigerant. In contrast, the total amount of water removed in the R600a experiment was slightly higher, which can be attributed to the higher initial product mass in the R600a experiment.

**Figure 14:**
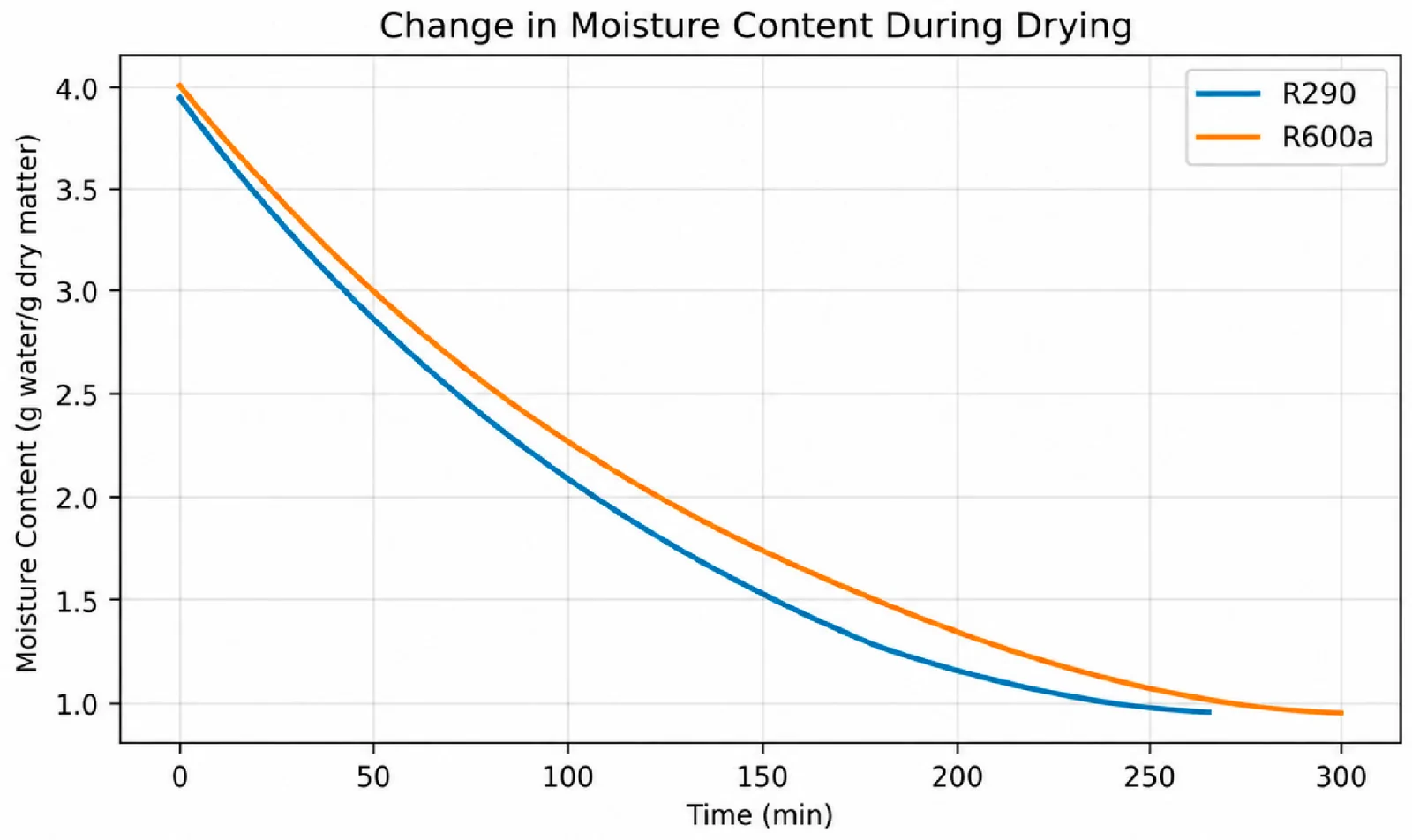
Time-dependent variation in MC_dry_ during the R290 and R600a drying experiments.

**Figure 15:**
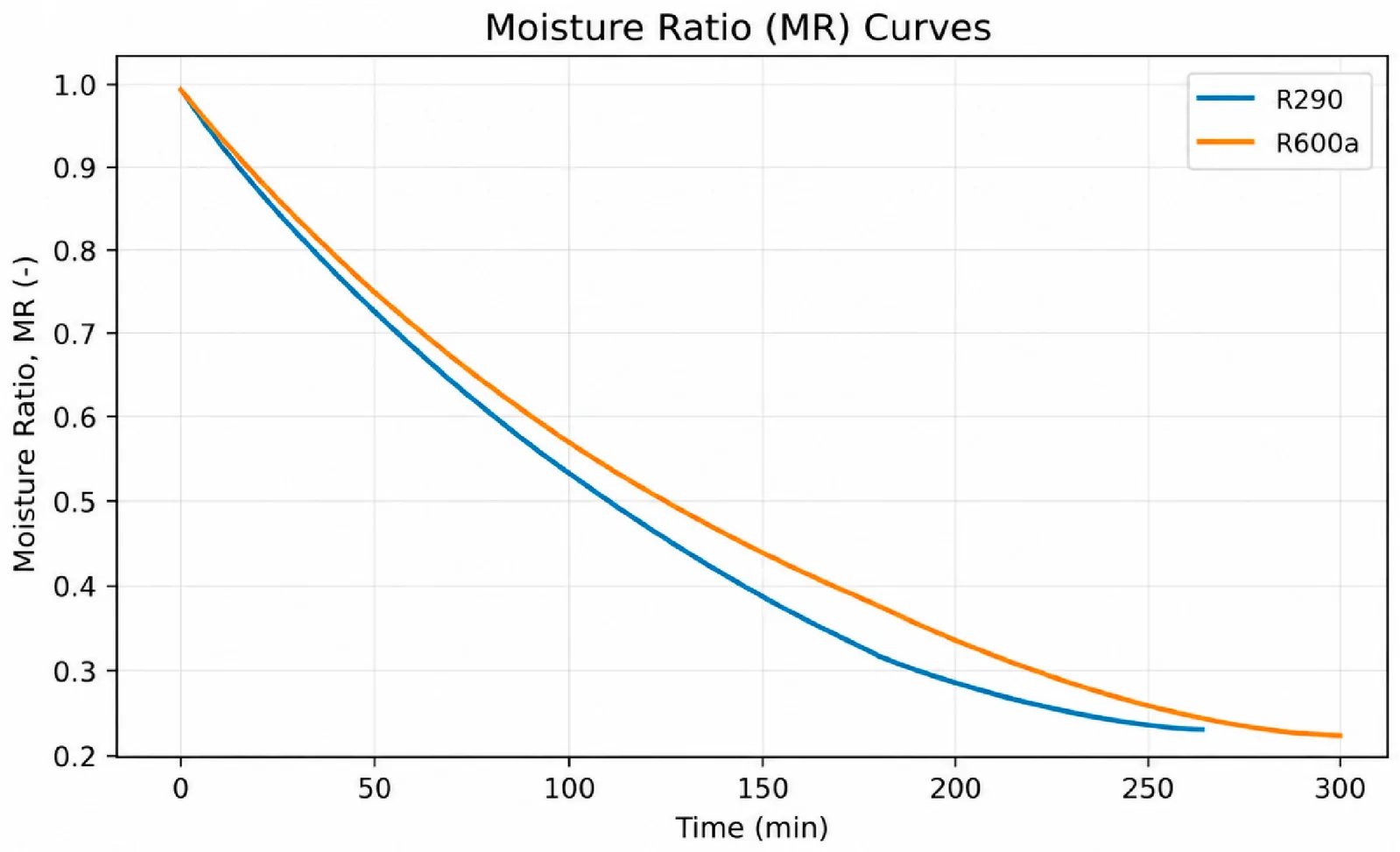
Variation in moisture ratio (MR) during the R290 and R600a drying experiments.

The MC_dry_ and MR curves presented in Figure 14 and Figure 15 show that the moisture content continuously decreased as drying progressed, while the downward trend of the curves was maintained throughout the experiments. The more rapid decrease in the MR value under the R290 condition supports the higher moisture removal rate observed with this refrigerant. In contrast, the R600a curve exhibited a longer drying period with lower energy consumption. At the end of the experiments, the MR values were calculated as 0.219 for R290 and 0.212 for R600a, indicating that both applications removed a substantial portion of the initial moisture content. The MC_dry_ and MR values at different stages of the drying process are presented in Table 10.

**Figure 16:**
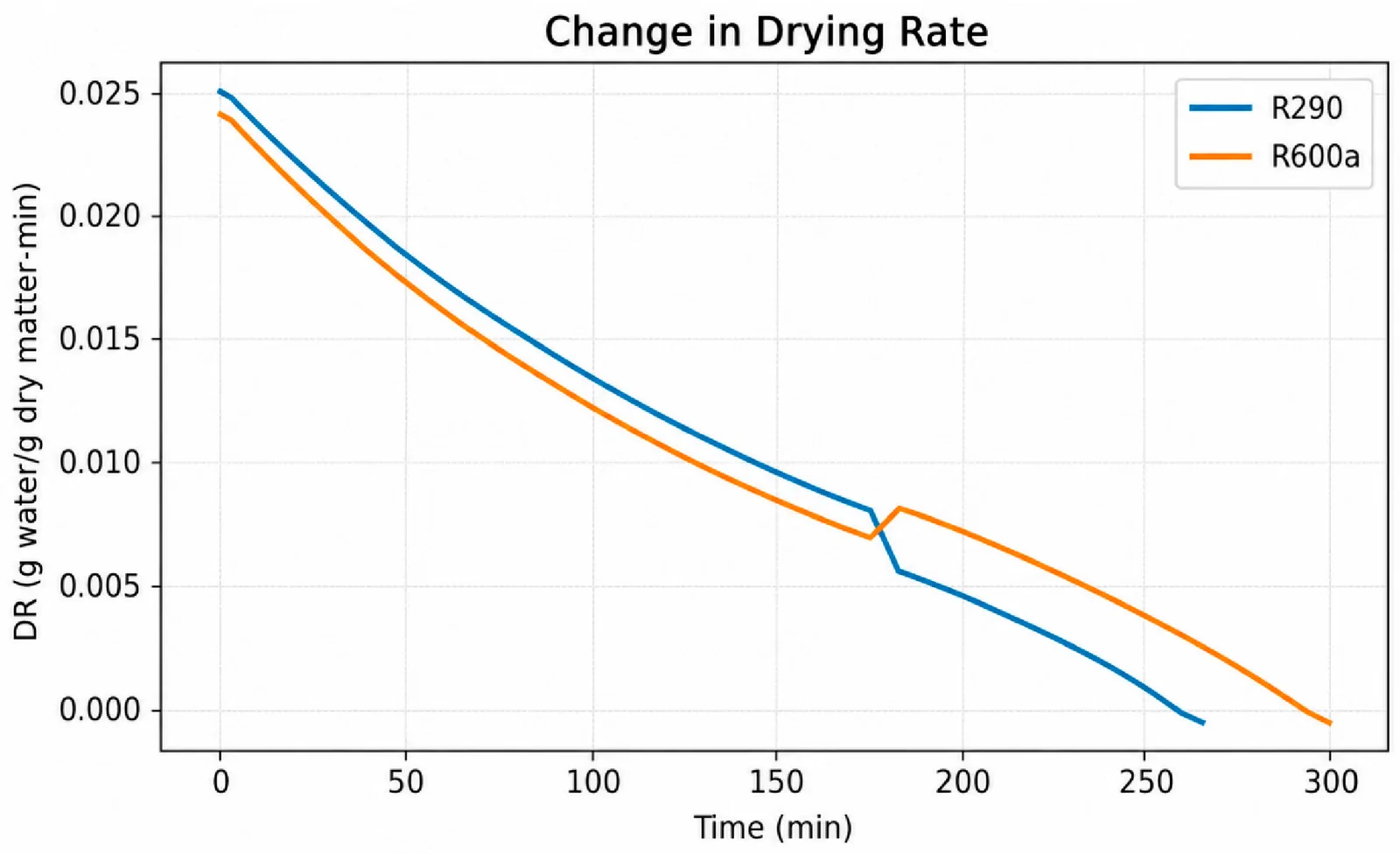
Variation in drying rate (DR) during the R290 and R600a drying experiments.

The drying rate curves given in Figure 16 show that the DR values gradually decreased with decreasing moisture content. The average DR values were determined as 0.01179 g water/g dry matter·min for R290 and 0.01075 g water/g dry matter·min for R600a. This finding indicates that moisture transport within the product and moisture removal from the surface were faster under the R290 condition. However, the advantage observed when considering only the drying rate should be evaluated together with the energy efficiency indicators.

### 3.3. Artificial Intelligence Findings

The experiments revealed a clear distinction in the physical strength profiles among the refrigerants. For R290, only T_10_ and T_8_ exhibited strong, well-discriminating, and monotonic signals. In contrast, all attributes in the R600a system varied over a wider range (Table 11). The calculated g_i_ physical strength values indicate that T_10_ and T_8_ were the dominant attributes for identifying the R290 refrigerant, while the effects of ambient humidity and secondary temperatures remained at negligible levels. In contrast, the high physical strength scores (g_i_ > 0.91) of all thermal and environmental parameters in the R600a system indicate that the PCI penalty mechanism appropriately preserves a broader combination of attributes for identifying this refrigerant.

The quality metrics of the Pareto fronts presented in Table 12 demonstrate that the model exhibited high convergence (HV ≥ 0.800) and a homogeneous distribution (Spacing ≤ 0.192) across all stages for R600a detection. The low HV and high standard deviation values observed for R290 (up to 0.369) are consistent with the search uncertainty caused by the narrow feature margins. The average front sizes (min. 3.7, max. 5.7) confirm that the method generated a rich Pareto set of alternative solutions without excessive convergence toward a single solution. This indicates that a practical portfolio of alternative rules was obtained at an optimal level, representing trade-offs among objectives in an explainable artificial intelligence (XAI) framework and allowing domain experts to readily evaluate the alternatives.

Table 13 presents the dominant rules generated by the proposed P-CRb-SPEA2 model for each “refrigerant, stage” pair with high PCI values, together with their Recall, ordinal error (OrdErr), PCI, and number of conditions. The model identified highly interpretable rule expressions that achieve maximum physical consistency (PCI = 1.0) and minimum rule complexity (conditions = 1) in both scenarios. For R290, all three stages are represented by threshold values of the T_10_ temperature attribute, whereas T_8_ emerges as the primary physical driver for R600a. In the R600a Early stage, the single-condition T_8_ rule achieved maximum sensitivity (Rec = 1.000), while maintaining a relatively high ordinal error (OrdErr = 0.745). In contrast, the alternative Early rule sacrificed some sensitivity (Rec = 0.903, PCI = 0.96) while reducing the ordinal error to zero, demonstrating the flexible trade-off capability offered by the multi-objective framework. Furthermore, as the drying stage progresses, the rule thresholds exhibit physically consistent trends, decreasing along the cooling direction for R290 based on T_10_ and increasing along the heating direction for R600a based on T_8_. This demonstrates that the model successfully learned not only mathematical correlations but also the underlying physical process dynamics.

Table 14 presents the results of the 10-fold stratified cross-validation (10 folds × 3 runs) conducted to evaluate the dependence of the proposed model on a single train/test split and to mitigate the potential risk of sampling bias. The Hypervolume (HV) values provide information on the average performance, while the min–max ranges and cross-fold standard deviation values demonstrate the robustness and generalizability of the model across different data partitions. The results indicate that the method exhibited highly robust performance for the R600a refrigerant, largely independent of fold distributions. High mean HV scores were obtained across all stages for R600a (min. 0.734, max. 0.901), while the cross-fold standard deviations remained relatively low (0.085–0.105). In particular, the mean HV of 0.901 ± 0.085 and maximum HV of 0.993 achieved in the Intermediate stage demonstrate the high robustness of the algorithm against dataset variations and different fold structures. In contrast, the mean HV scores for R290 (min. 0.467, max. 0.697) were lower and exhibited greater cross-fold variability. The wide range between the minimum (0.208) and maximum (0.990) HV values indicates that the performance of R290 detection is more dependent on the fold distribution. The overlap between cross-fold performance and single-split results further confirms that the observed differences in model quality are not merely a consequence of random sampling but rather reflect a structural characteristic arising from the feature ranges of the refrigerants.

The performance results of conventional machine learning models, namely Naive Bayes (NB), k-NN, Support Vector Machines (SVM), Decision Tree (DT), and Random Forest (RF), under 10-fold stratified cross-validation are presented in Table 15. The results show that the tree-based and neighborhood-based methods (RF, DT, and k-NN) achieved highly comparable and, in some cases, very high-performance levels for both refrigerants. While Naive Bayes (NB) maintained an acceptable performance level, the SVM model exhibited relatively unstable performance, mainly due to its sensitivity to scaling. This finding indicates that, based solely on accuracy-oriented performance metrics, statistical superiority among conventional methods cannot be conclusively established; therefore, claiming model superiority solely based on accuracy rates would not be methodologically justified. In addition, the proposed P-CRb-SPEA2 method provides advantages not offered by conventional models, particularly in terms of PCI and a high degree of interpretability, while maintaining comparable performance levels. Thus, the proposed method stands out by providing flexible rule sets that are not limited to accurate predictions but are also fully consistent with the physical process and can be readily validated by domain experts.

Table 16 comparatively presents the methodological characteristics and structural capabilities of the proposed P-CRb-SPEA2 approach and conventional classification algorithms. Conventional models focus primarily on a single dominant prediction output and treat the physical constraints and ordinal relationships among classes as secondary considerations. Although a Decision Tree model can generate interpretable rule structures with 5–7 nodes and can capture some physical relationships, it lacks multi-objective optimization capability. In contrast, the proposed P-CRb-SPEA2 algorithm maintains model complexity at a minimum by generating highly interpretable rules with very sparse structures (1–2 conditions). The method directly incorporates physical awareness into the model through the PCI, explicitly optimizes ordinal errors through the OrdErr metric, and provides flexible trade-off alternatives to the decision-maker with an average Pareto set size ranging from 3.7 to 5.7. These characteristics demonstrate that the proposed method provides a distinct methodological and practical advantage over conventional black-box and single-objective models.

## 4. Conclusions

In this study, the natural refrigerants R290 and R600a were experimentally compared in a vapor-compression refrigeration system, and the condenser waste heat was utilized for apple drying. The refrigeration tests were conducted at an evaporator temperature of −16 °C and condenser temperatures of 25, 30, 35, and 40 °C under identical operating conditions for both refrigerants, and the drying performance was evaluated in terms of drying kinetics and energy indicators. In addition, a physics-guided multi-objective evolutionary algorithm, P-CRb-SPEA2, was developed to identify the drying stages from thermal and environmental measurements without continuous product weighing. The key results obtained are summarized as follows:

- R290 provided 73% higher cooling capacity, 59% greater condenser heat rejection ($\dot{Q}$_cond_), and approximately 15.7% higher COP_HP_ than R600a, indicating its advantage in terms of thermodynamic performance and the utilization of condenser waste heat for drying.
- R290 reduced the drying time from 300 to 265 min and provided a higher average drying rate and effective moisture diffusivity (1.007 × 10^−9^ m^2^/s for R290 and 8.967 × 10^−10^ m^2^/s for R600a), confirming faster internal moisture transport.
- R600a showed better energy performance during drying, with a lower specific energy consumption and a higher specific moisture extraction rate. Therefore, R290 was more favorable for drying intensity and process duration, whereas R600a was more advantageous when energy efficiency was prioritized.
- The P-CRb-SPEA2 approach classified the drying process into ordered stages by considering recall, ordinal error, physical consistency, and model complexity. T_10_ was the dominant process indicator for R290, whereas T_8_ played the leading role for R600a.
- Ten-fold cross-validation showed that the proposed method maintained robust performance while providing physically consistent and interpretable rules, which conventional machine-learning models do not explicitly offer, and demonstrated that drying stages can be identified without relying solely on continuous product weighing.

Overall, the study demonstrates that an integrated refrigeration–drying system can simultaneously provide useful cooling and recover condenser waste heat for food drying. At the same time, explainable artificial intelligence can add an intelligent process-monitoring layer to the system. The combined experimental, drying, and XAI results indicate that refrigerant selection should be based on a multi-criteria assessment involving thermodynamic performance, drying kinetics, energy efficiency, and process interpretability.

## Figures and Tables

**Table 1:** Summary of the main studies reviewed and their key findings.

Reference	System/Method	Key Findings
Sánchez et al. [7]	Beverage cooler; R290 and R600a vs. R134a	Both R290 and R600a consumed less power than R134a.
Rasti et al. [8]	Domestic refrigerator; R600a vs. R134a	Power consumption decreased by 7% with an HFC-type compressor and by 18.7% with an HC-type compressor.
Berkah Fajar et al. [9]	Small vapor-compression system; R290 vs. R410A	The highest COP was 4.9 with R290, compared with 4.6 with R410A.
Sánchez et al. [10]	Refrigeration facility; R290 vs. R134a	R290 provided higher COP values at all evaporator and condenser temperatures.
Patel and Kar [11]	Heat pump-assisted drying of agricultural products	Lower energy consumption and better moisture control than conventional drying methods.
Liu et al. [12]	Air-source heat pump dryer for food	The thermal performance of the system plays a determining role in drying efficiency.
Menlik et al. [13]	ANN; freeze-drying of apples	MC, MR, and DR were successfully predicted from drying time, pressure, sample thickness, chamber and product temperatures, and relative humidity.
Keramat-Jahromi et al. [14]	RF and k-NN with online image features; date fruit	MR was predicted with test R^2^ values of 0.976 (RF) and 0.959 (k-NN).
Sharabiani et al. [15]	ANN, ANFIS, and SVR	MR was predicted from drying time, air temperature, and infrared power; SVR provided particularly high predictive accuracy.
[16,17,18]	ANN and SVM	MR prediction and process optimization under different drying conditions.

**Table 2:** Characteristics of sensors.

Instrument	Type	Range	Brand	Accuracy
Pressure sensors	Pressure transmitter	0 to 100 bar	DANFOSS	±0.3%
Temperature sensors	PT100	−50 °C to +230 °C	REISSMANN	±0.5 °C
Flow meter	Turbine	0.1 to 10 L min^−1^	TEKSENS	±0.1%
Wattmeter	Single phase energy meter	0 to 4000 W	ENTES	±0.1%

**Table 3:** Experimental operating conditions applied to both R290 and R600a.

Operational Conditions	Values
Evaporator temperature (°C)	−16
Condenser temperatures (°C)	25, 30, 35, 40

**Table 4:** Uncertainty analysis of measurements.

Sensor	Uncertainty Calculation
Temperature	±0.7 °C
Flow rate	±0.5%
Pressure	±0.02 bar
Compressor power consumption	±0.1%

**Table 5:** Information related to drying experiments.

Parameters	R290	R600a	Unit
Initial product mass, W_0_	170.0	173.0	g
Final product mass, W_f_	63.8	63.4	g
Dry mass, W_d_	34.0	34.0	g
Amount of water removed	0.1062	0.1096	kg
Drying time	265	300	min
Average drying temperature	313	300	K
Total energy consumption	1.850	1.000	kWh
Product half-thickness, L	0.005	0.005	m

**Table 6:** Determining TP, TN, FP, and FN counters in rule evaluation.

IF Part	THEN Part	Counter
True	True	True Positive (TP)
True	False	False Positive (+ordinal penalty) (FP)
False	True	False Negative (FN)
False	False	True Negative

**Table 7:** R290 and R600a dataset.

Gas	Number of Data	Early Class	Intermediate Class	Advanced Class	Attribute
R290	266	80	101	85	6
R600a	301	88	104	89	6

**Table 8:** Ordinal drying-stage classes.

Stage/Class	Condition
Early	0.60 < MR ≤ 1.00
Intermediate	0.30 < MR ≤ 0.60
Advanced	0.00 ≤ MR ≤ 0.30

**Table 9:** Basic moisture and mass results for the R290 and R600a drying experiments.

Refrigerant	W_0_ (g)	W_f_ (g)	Time (min)	Water Removed (kg)	MC_wet,i_ (%)	MC_wet,f_ (%)	MC_dry,i_	MC_dry,f_	MR_f_
R290	170.0	63.8	265	0.1062	80.00	46.71	4.000	0.876	0.219
R600a	173.0	63.4	300	0.1096	80.35	46.37	4.088	0.865	0.212

**Table 10:** MC_dry_ and MR values at different drying time ratios.

Time Rate	R290 min	R290 MR	R290 MC_dry_	R600a min	R600a MR	R600a MC_dry_
0% drying time	0	1.000	4.000	0	1.000	4.088
25% drying time	66	0.654	2.617	75	0.643	2.630
50% drying time	132	0.421	1.685	150	0.424	1.735
75% drying time	199	0.273	1.091	225	0.278	1.136
100% drying time	265	0.219	0.876	300	0.212	0.865

**Table 11:** Calculated physical strength values of features for target gases.

Gas	T_10_	T_3_	T_7_	RH_Ambient_	T_8_	T_2_
R290	1.000	0.057	0.192	0.004	0.996	0.455
R600a	0.982	0.992	0.914	0.938	1.000	0.979

**Table 12:** Multi-objective quality indicators (30 run, mean ± standard).

Gas	Stage/Class	HV	IGD	Spacing	Avr. Pareto Fr. Size
R290	Early	0.513 ± 0.314	0.487 ± 0.310	0.277	4.6
R290	Intermediate	0.696 ± 0.260	0.322 ± 0.234	0.226	5.1
R290	Advanced	0.564 ± 0.369	0.450 ± 0.370	0.202	3.7
R600a	Early	0.800 ± 0.171	0.239 ± 0.142	0.192	4.8
R600a	Intermediate	0.927 ± 0.090	0.125 ± 0.081	0.125	5.7
R600a	Advanced	0.868 ± 0.197	0.155 ± 0.187	0.106	4.9

**Table 13:** The highest PCI rule for each “gas, stage”.

Gas	Stage/Class	Rec	OrdErr	PCI	Cond.	Rule
R290	Early	0.893	0.000	1.0	1	IF (−9.567 < T_10_ < −8.900) THEN Early
R290	Intermediate	0.986	0.017	1.0	1	IF (−10.474 < T_10_ < −9.685) THEN Intermediate
R290	Advanced	0.966	0.000	1.0	1	IF (−10.900 < T_10_ < −10.492) THEN Advanced
R600a	Early	1.000	0.745	1.0	1	IF (28.700 < T_8_ < 32.196) THEN Early
R600a	Early	0.903	0.000	0.96	2	IF (28.5 < T_3_ < 32.7) AND (16.5 < RH_Ambient_ < 16.9) THEN Early
R600a	Intermediate	0.586	0.040	1.0	1	IF (30.872 < T_8_ < 31.991) THEN Intermediate
R600a	Advanced	0.629	0.000	1.0	1	IF (32.097 < T_8_ < 32.816) THEN Advanced

**Table 14:** Hypervolume performance across 10-fold stratified cross-validation.

Gas	Stage/Class	HV	HV min	HV max
R290	Early	0.479 ± 0.181	0.208	0.704
R290	Intermediate	0.697 ± 0.187	0.525	0.990
R290	Advanced	0.467 ± 0.269	0.248	0.927
R600a	Early	0.734 ± 0.096	0.593	0.862
R600a	Intermediate	0.901 ± 0.085	0.763	0.993
R600a	Advanced	0.874 ± 0.105	0.719	1.000

**Table 15:** Performance comparison of classical machine learning models under 10-fold stratified cross-validation (mean ± SD).

Gas	Model	Accuracy	Macro-F1	OrdMAE
R290	NB	0.899 ± 0.068	0.898 ± 0.072	0.101 ± 0.068
R290	k-NN	0.993 ± 0.015	0.993 ± 0.015	0.007 ± 0.015
R290	SVM	0.380 ± 0.011	0.183 ± 0.004	0.620 ± 0.011
R290	DT	0.993 ± 0.015	0.993 ± 0.015	0.007 ± 0.015
R290	RF	0.996 ± 0.011	0.996 ± 0.011	0.004 ± 0.011
R600a	NB	0.953 ± 0.031	0.955 ± 0.029	0.047 ± 0.031
R600a	k-NN	0.990 ± 0.015	0.990 ± 0.015	0.010 ± 0.015
R600a	SVM	0.648 ± 0.051	0.529 ± 0.046	0.352 ± 0.051
R600a	DT	0.993 ± 0.013	0.994 ± 0.013	0.007 ± 0.013
R600a	RF	0.993 ± 0.013	0.994 ± 0.013	0.007 ± 0.013

**Table 16:** Methodological comparison of classification models in terms of complexity, physics awareness, and trade-off optimization.

Model	Model Complexity	Physical Awareness	Number of Solutions	Ordinal Optimization	Trade-Off
NB	Parametric	-	Single	-	-
k-NN	Complete dataset	-	Single	-	-
SVM	Support vectors	-	Single	-	-
DT	5–7 nodes	-	Single	-	-
RF	100 trees	Indirect	Single	-	-
P-CRb-SPEA2	1–2 condition	✓	Multiple (3.7–5.7)	✓	✓

## Data Availability

The data supporting the findings of this study are not publicly available.

## Abbreviations

The following abbreviations are used in this manuscript:

COP	coefficient of performance
GWP	global warming potential
h	enthalpy (kJ kg^−1^)
$\dot{m}$	mass flow rate (kg s^−1^)
P	pressure (bar)
PLC	programmable logic controller
$\dot{Q}$	heat transfer rate (W)
T	temperature (°C)
W	compressor power consumption (W)
cond	condenser
comp	compressor
dis	discharge
evap	evaporator
HP	heat pump
i	inlet
o	outlet
R	refrigeration
